# Engineering Dehalogenase Enzymes Using Variational Autoencoder-Generated Latent Spaces and Microfluidics

**DOI:** 10.1021/jacsau.4c01101

**Published:** 2025-02-13

**Authors:** Pavel Kohout, Michal Vasina, Marika Majerova, Veronika Novakova, Jiri Damborsky, David Bednar, Martin Marek, Zbynek Prokop, Stanislav Mazurenko

**Affiliations:** † Loschmidt Laboratories, Department of Experimental Biology and RECETOX, Faculty of Science, Masaryk University, Brno 611 37, Czech Republic; ‡ International Clinical Research Centre, St. Anne’s Hospital, Brno 656 91, Czech Republic

**Keywords:** dehalogenase, protein engineering, machine learning, microfluidics, protein stability, variational autoencoder

## Abstract

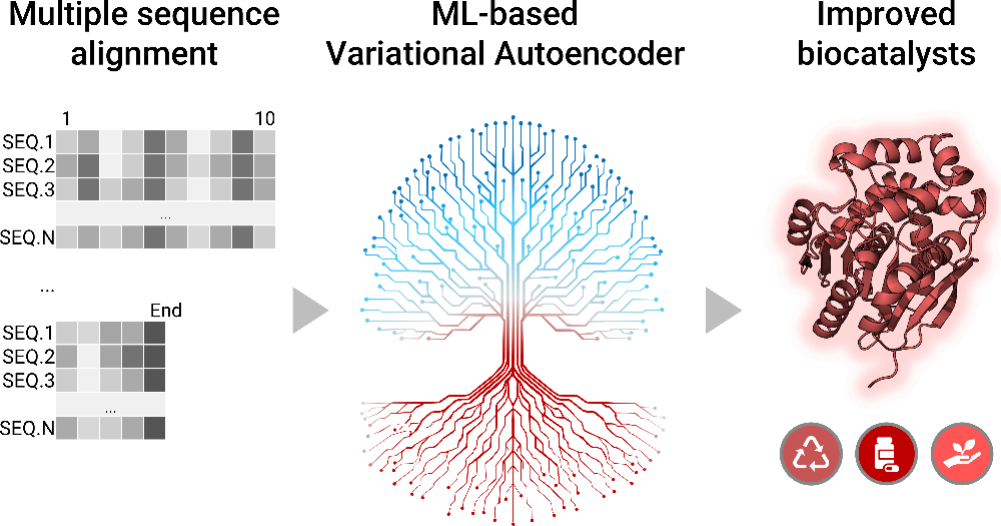

Enzymes play a crucial role in sustainable industrial applications, with their optimization posing a formidable challenge due to the intricate interplay among residues. Computational methodologies predominantly rely on evolutionary insights of homologous sequences. However, deciphering the evolutionary variability and complex dependencies among residues presents substantial hurdles. Here, we present a new machine-learning method based on variational autoencoders and evolutionary sampling strategy to address those limitations. We customized our method to generate novel sequences of model enzymes, haloalkane dehalogenases. Three design–build–test cycles improved the solubility of variants from 11% to 75%. Thorough experimental validation including the microfluidic device MicroPEX resulted in 20 multiple-point variants. Nine of them, sharing as little as 67% sequence similarity with the template, showed a melting temperature increase of up to 9 °C and an average improvement of 3 °C. The most stable variant demonstrated a 3.5-fold increase in activity compared to the template. High-quality experimental data collected with 20 variants represent a valuable data set for the critical validation of novel protein design approaches. Python scripts, jupyter notebooks, and data sets are available on GitHub (https://github.com/loschmidt/vae-dehalogenases), and interactive calculations will be possible via https://loschmidt.chemi.muni.cz/fireprotasr/.

## Introduction

Biocatalysis is a promising field that offers sustainable and environmentally friendly solutions for industries increasingly driven by enzymes. Thanks to millions of years of evolution, enzymes are fine-tuned to carry out specific chemical reactions with high efficiency. This makes them attractive alternatives to traditional catalysis, which often relies on harsh conditions and toxic chemicals.^1^ Thus, these biocatalysts find application across various industries, including pharmaceuticals, food production, and sustainability efforts aimed at reducing waste and energy consumption.^2^ Since natural enzymes often exhibit suboptimal performance in non-native environments, enzyme engineering is usually required to unlock their full potential.^3,4^ In addition to commonly used experimental approaches such as directed evolution, scientists can also expedite the process and reduce associated development costs by incorporating computational methods.^5,6^ One group of computational methods rely on physical-based modeling techniques such as Empirical Valence Bond (EVB)^7−9^ and hybrid Quantum Mechanics/Molecular Mechanics (QM/MM) methods, which simulate atomic-level interactions and energy landscapes of enzymes.^10,11^ Another group of methods exploit protein sequences. These methods help navigate the vast sequence space, as it is estimated that only a fraction of all possible sequences fold into functional protein structures.^12^ Most natural proteins have marginal stability,^13^ thus posing a significant risk for any manipulations with their sequences.

Many computational methods aiming to refine the search space of such sequence manipulations rely on homologous sequences.^14,15^ These sequences of different but related proteins stemming from a common ancestor contain rich evolutionary information.^16^ Homologous protein sequences can be employed to identify conserved and functionally important regions, suggest beneficial mutations, and create phylogenetic trees.^17^ Notable examples of approaches in this context include the Maximum Entropy (MaxEnt) model and ancestral sequence reconstruction. The MaxEnt model employs statistical energy derived from homologous sequences, applying the maximum entropy principle to establish correlations with enzyme catalysis and stability in both the active site and more distant regions.^18,19^ Ancestral sequence reconstruction utilizes phylogenetic trees and sequence alignment techniques to trace evolutionary changes and infer the sequences of ancestral proteins. This method has proven to be a promising strategy for enhancing protein stability.^20−24^

Despite the recent progress in extracting evolutionary information from multiple sequence alignments (MSA) of homologous proteins, analyzing this variability is challenging. Historically, this data was used primarily by looking at only one or two positions at a time.^25,26^ More recent approaches extract patterns by deep neural networks, in particular algorithms that map the sequence space onto their internal low-dimensional representation, also referred to as latent spaces. Generative models trained on large data sets of tens of thousands of sequences have shown excellent results in producing highly interpretable embeddings and generating novel protein variants.^27−30^ The most recent examples of this class include diffusion models, which are trained to denoise synthetically noised inputs. They were initially used to generate protein backbone structures^31,32^ and predict the binding of a flexible ligand to a protein^33^ but later have been adapted to generating sequences as well.^34,35^ Another example is Generative Adversarial Networks, which learn to generate new data through competitive training involving two artificial neural networks. For instance, ProteinGAN was used to generate functional protein sequences of malate dehydrogenases.^36^ Variational autoencoders (VAEs) are a third type of MSA-based models, which shows a particular promise in this domain due to the explicit modeling of the latent space.^37^ VAEs have already proven useful in several applications, including predicting protein structures,^38^ discovering novel drugs,^39^ and predicting protein functions.^40^ By learning the latent space representation of a specific family, VAEs provide valuable insights into the evolution of protein families, as demonstrated in recent studies exploring the phylogenetic relationships within the latent space.^41−43^ In particular, Ding et al. showed that the latent space of the variational autoencoders can capture the biophysical properties of protein variants and the phylogenetic relationships within protein families.^41^ However, the study did not offer a strategy that would allow exploiting these relationships to generate new proteins from the latent space. For a comprehensive overview of generative models, we refer the reader to an excellent recent review.^44^

**Figure 1:**
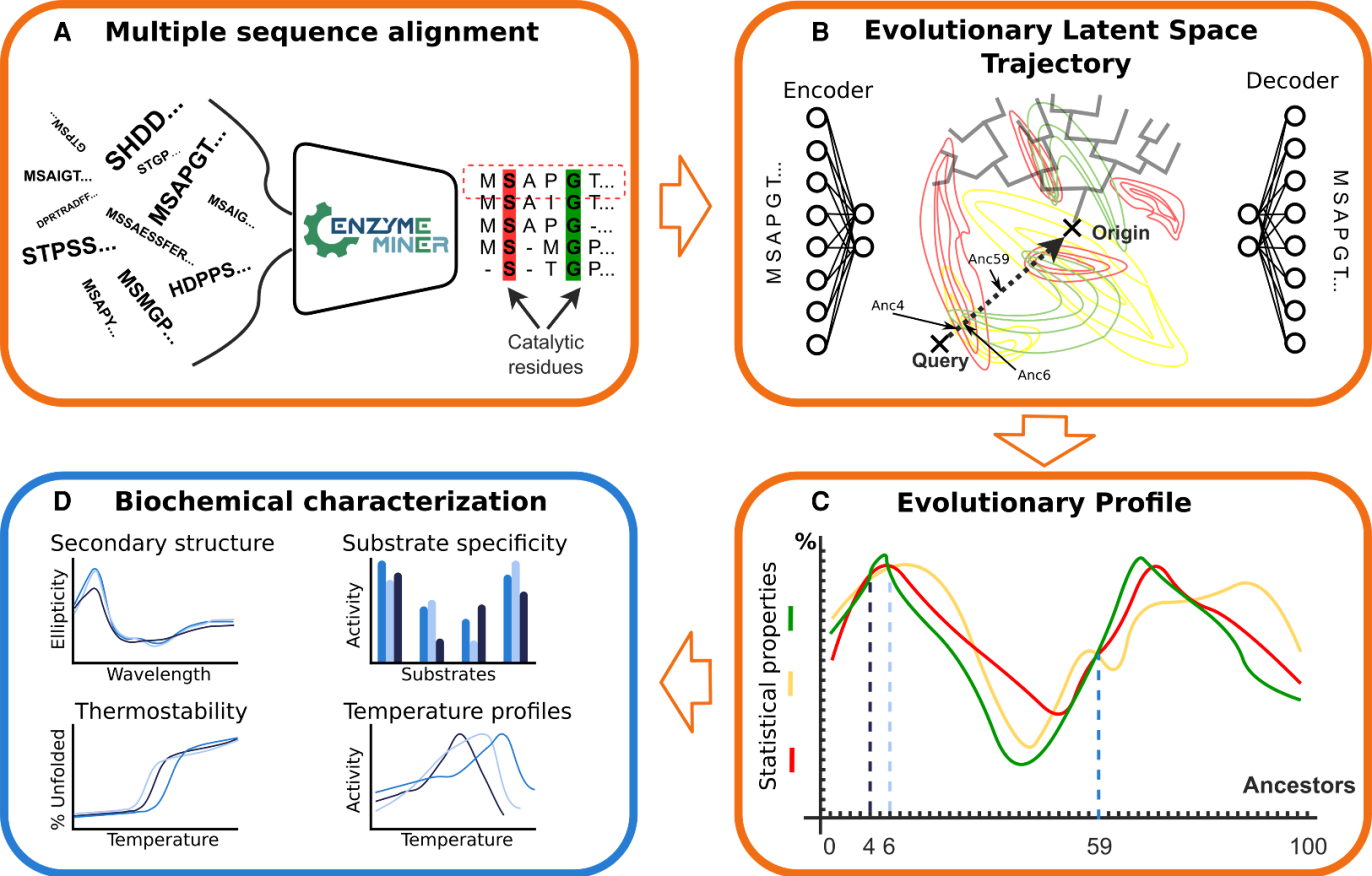
The scheme of the variational autoencoder-based pipeline for the design of novel sequences. (**A**) Advanced sequence search of homologous proteins using EnzymeMiner.^47^ (**B**) Optimization of the variational autoencoder architecture to capture the sequence distribution of the MSA and phylogenetic dependencies within the latent space. (**C**) Exploration of the evolutionary dependencies between the sequences extracted from the variational autoencoder and its low-dimensional latent space. This representation is then used to guide the protein design strategy and generate sequences along the trajectory from the query to the latent space origin. The generated sequences are characterized based on their statistical and sequential properties to produce the evolutionary profile. This profile serves as a guide for selecting designs. (**D**) The experimental characterization of the proposed designs is conducted. The orange frames represent the computational steps and the blue frame is the experimental step.

Here we propose a simple strategy to leverage the evolutionary-shaped geometry of the VAE-learned latent space to design novel ancestral-like variants of haloalkane dehalogenases (HLDs; EC 3.8.1.5). These enzymes cleave the carbon–halogen bonds^45^ and are widely used in biocatalysis, biosensing, cell imaging, and protein analysis.^46^ The proposed workflow is based on a small number of proteins with known functions and aims to produce new variants that preserve catalytic function and improve stability (Figure 1). First, we mined sequences with preserved catalytic residues using EnzymeMiner^47^ to obtain an MSA of functionally related proteins. Second, we trained a VAE and specified several metrics to measure its capacity to generate protein sequences and capture the phylogeny in the constructed latent representations. Third, based on the geometry of the latent space, we developed a sampling strategy and produced a statistical profile of candidate sequences to select promising variants from the evolutionary trajectory. Fourth, we overexpressed and characterized variants experimentally using advanced microfluidics. Three consecutive rounds of experimental characterization and workflow optimization resulted in 20 variants, sharing as little as 67% sequence similarity to known HLDs. Obtained enzymes showed up to a 9 °C increase in melting temperatures and an average improvement of 3 °C across all soluble variants. We also observed a boost in activity, up to 3.5-fold for the most stable variant, whereas most of the other expressed variants showed activity levels comparable to benchmark enzymes.

## Materials and Methods

### MSA and Data Preprocessing

Two data sets, HLDI-IV, and HLDI-II, were created using the EnzymeMiner tool^47^ based on haloalkane dehalogenase sequences. Both data sets underwent preprocessing, including sequence filtering, gap reduction, and clustering, to ensure diversity. The second data set also underwent additional adjustments to reduce gaps and improve solubility rates. Detailed descriptions of the preprocessing steps and data set creation are provided in SI Section 1.1. These data sets were then used to train models in multiple experimental rounds, specifically HLDI-IV for rounds I and II, and HLDI-II for round III.

Two sets of experimentally measured stability values were mapped to the latent space of VAE Model 1. The first set consisted of six ancestral sequences from the previous ancestral campaign of the thoroughly characterized dehalogenases DbjA, DbeA, DhaA, DmxA, and DmmA.^48^ These sequences were realigned with the original input MSA and preprocessed accordingly. The second set consisted of 24 previously engineered DhaA variants based on the FireProt method,^22,49,50^ similarly aligned and preprocessed with the query sequence P59336_S14 of the input MSA.

### Variational Autoencoders and Training

Variational autoencoders (VAEs)^37^ are a type of deep generative learning model whose goal is to learn the data distribution. VAEs consist of two main components: an encoder and a decoder (Figure 1B). The encoder takes the input sequence and maps it to a lower dimensional representation called a latent space. Within this latent space, the encoded input is modeled as a normal distribution by two parameters, the mean and the variance. Subsequently, the decoder draws samples from this latent space distribution and maps these samples back to sequences. The training of VAEs is based on minimizing the loss function made of the reconstruction term (penalizes incorrect reconstruction of the input data) and the regularization term (serves to constrain the latent space distribution of encoded values). The latter forces the latent space to be close to normal distribution by measuring the Kullback–Leibler divergence. As a result, the individual distributions are forced to overlap within the latent space, ensuring proper alignment of the sequences corresponding to the nearby points in the latent space. We tested several architectures and eventually used one hidden layer in the encoder and decoder, both composed of N neurons, where N is the width of preprocessed MSA (number of positions), the latent space dimensionality of 2, and either zero (rounds I and II) or decreasing (round III) weight decay. We employed the tanh activation function without dropout and assigned equal weighting to the reconstruction and regularization terms in the training objective (SI Section 1.4). The final model had 3 million parameters. We used the Adam optimizer with a learning rate of 0.001 and stopped training after not improving the loss function for more than 3 consecutive rounds.

In the conditional variational autoencoders (CVAEs),^51^ a tag (LOW, MEDIUM, HIGH) was added to the encoder and decoder to represent different solubility levels. The tags were generated based on the solubility values predicted by SoluProt^52^ and binned to achieve uniform distribution across bins and ensure balanced sample extraction.^53^ A detailed description can be found in SI Section 4.

### Model Generative Capacity

We performed a first- and second-order statistical analysis to compare the frequency of amino acids at each position in the multiple sequence alignment (MSA) between input and generated data sets. First-order statistics assess the occurrence of each amino acid at a given position, while second-order statistics capture pairwise relationships between two positions. We computed the pairwise covariance scores to evaluate how well the generative model reproduces interactions between amino acids, an essential indicator for the likely stability and function of the generated proteins.^54^ For this study, we used 3,000 randomly selected samples from both input and generated data sets for the statistical comparisons. A detailed description is provided in SI Section 1.2.

### Average Reconstruction Accuracy and Controls

The average reconstruction accuracy of each sequence was approximated as an average reconstructed sequence identity for 5,000 samples around the original sequence coordinates of its latent space embedding based on the mean and variance returned by the encoder for a given sequence. The negative control subset was generated by sampling sequences from the profile of the input MSA only based on the amino acid frequencies in each position. The positive control subset comprised 5% of preprocessed sequences randomly selected from the MSA and excluded from training. Finally, the ancestral subset was composed of 100 reconstructed sequences by the straight evolutionary strategy (see Construction of the evolutionary trajectory). Except for the ancestral subset, all the subsets contained the number of sequences corresponding to 5% of the preprocessed data set.

### Phylogeny Mapping and Evaluation

We generated 13 phylogenetic trees using our input MSA to analyze the relationship between phylogenetic branches and the latent space. Each tree had around 100 randomly sampled nodes, and ancestral sequences were reconstructed using FireProtASR.^22^ We explored the correlation between the depth of nodes and their latent space embeddings and analyzed the directionality of tree branches within the latent space similar to.^41^ See SI Section 1.3 for more details.

### AlphaFold Structure Prediction and Manual Analysis of the Suggested Mutations

For structural predictions of ancestral sequences, the AlphaFold2 Google Colaboratory implementation, ColabFold, using MMseqs2, was used.^55^ We predicted structures without providing templates, and we performed amber relaxation with 200 steps on the top-ranked structure. We used the default MSA options with pair sequences from the same species and unpaired sequences from separate MSA for each chain (paired+unpaired option). The optimal structure was selected automatically by ColabFold. The refinement process was repeated over three cycles to improve the structure’s accuracy. The relaxed first-ranked structure was used as the result of the prediction.

In round 3, the proposed mutations by VAEs were also curated manually (see SI Section 2 for more detail). The visual inspection of the modeled AlphaFold variants was performed by Pymol,^56^ and the MutCompute web server^57^ was used to calculate the score per residue (log-likelihood ratio). Thus, a positive score indicates that MutCompute assesses the substituted residue as more likely to occur in the given structural microenvironment than the wild-type residue.

### Protein Production, Purification and Whole-Cell Activity Screening

First,*E. coli*BL21(DE3) cells (NEB, USA) were transformed with the pET21b expression plasmid containing the corresponding gene, plated on LB-agar with 100 μg/mL ampicillin, and incubated at 37 °C overnight (12–16 h). Cells transformed with pET21b::DhaAwt, pET21b::RLuc, and empty pET21b served as controls. For small-scale protein overexpression and affinity purification, cultivation in 96-deep well plates, harvesting, SDS-PAGE analysis, and high-throughput affinity purification using TALON SuperFlow Metal Affinity Resin (Takara) were performed (see details in SI Section 7.1). Cell cultivations for enzymatic screenings and halide oxidation (HOX) assay^58^ were carried out, including cell cultivation, harvesting, and whole-cell activity screening (see details in SI Section 7.2). For large-scale protein overexpression and purification, selected mutant enzymes were expressed in*E. coli*BL21(DE3), and purification was done using metal affinity resin and gel filtration (see details in SI sections 7.3–7.5).

### Secondary Structure Experimental Validation

The secondary structure of the analyzed variants was experimentally verified using circular dichroism (CD) spectroscopy, measured at 15 °C using a spectropolarimeter Chirascan (Applied Photophysics). The samples were dissolved in 1 mM HEPES buffer or in the 50 mM Phosphate buffer, and their concentration was adjusted to ∼0.18 mg/mL. Data were collected from 185 nm to 260 nm with 0.25 s integration time and 1 nm bandwidth using a 0.1 cm quartz cuvette. Each spectrum was obtained as an average of five individual repeats. Prediction of CD spectra was performed by PDBMD2CD^59^ (https://pdbmd2cd.cryst.bbk.ac.uk), using either experimental structures from PDB database (1CQW for DhaA) or AlphaFold models. The estimation of secondary structure elements from experimental data and PDB database structures was performed additionally by BeStSel^60^ (https://bestsel.elte.hu/).

### Thermal Denaturation by CD and NanoDSF

Thermal unfolding of selected enzyme variants was carried out using a Chirascan spectropolarimeter (Applied Photophysics, UK). Each protein sample was diluted in 50 mM Phosphate buffer to the concentration of 0.18 mg·mL^–1^ and measured in a 0.1 cm quartz cuvette. Changes of ellipticity were monitored at three wavelengths (195 nm, 210 nm, and 227 nm) from 15 to 80 °C with a 0.1 °C resolution and 1 °C·min^–1^ heating rate. Recorded data were fitted using the model “Sigmoid curve + slope” in the Pro Data Viewer software (Applied Photophysics, UK). The apparent melting temperature (*T*_m^app^_) was evaluated as a midpoint of the normalized thermal transition.

Thermal unfolding was further studied using NanoDSF Prometheus NT.48 (NanoTemper, Germany) by monitoring tryptophan fluorescence over the temperature range of 20 to 95 °C, at a heating rate of 1 °Cwith 20% excitation power. The thermostability parameters (*T*_on_ and *T*_m^app^_) were evaluated directly by ThermControl v2.0.2.

### Dehalogenase Activity Measurements on MicroPEX

Activity measurements for the determination of temperature profiles and substrate specificity were conducted on the capillary-based droplet microfluidic platform MicroPEX,^61^ enabling the characterization of specific enzyme activity within droplets for multiple enzyme variants in one run. A detailed description of the microfluidic method can be found elsewhere^62,63^ and briefly in SI Section 7.6.

## Results

We developed the pipeline to leverage the power of the variational autoencoder and its latent spaces for the design of promising biocatalysts (Figure 1). This pipeline was inspired by the previous studies reporting the connections between latent space geometry and phylogeny for a given MSA,^41,43^ however, this connection has not been exploited for generating new protein sequences thus far. We hypothesized that the coordinates within the latent space could serve as a navigational tool for identifying ancestral-like sequences, offering a way to improve the stability of query proteins while maintaining their function. We iteratively executed our pipeline across three rounds, each iteration followed by experimental validation to improve our workflow (SI Section 2). In both the first and second rounds, we utilized the same trained VAEs (Model 1), with the only difference being a revised selection of VAEs ancestors. In the third round, we introduced changes to the MSA preprocessing and additionally manual curation of the generated ancestors by AlphaFold and MutCompute.^16,57^ In addition, during the third round of experimental validation, we explored the possibility of conditioning the VAEs on solubility scores returned by the ML-based tool SoluProt.^52^ In total, three trained VAEs models were explored in the third round (Models 2–4) to better understand the strengths and weaknesses and refine our approach.

### Multiple Sequence Alignment Processing

#### The Data Collection Is Optimized to Preserve Catalytic Activities

The first step of our pipeline is to construct an MSA. Instead of using Pfam alignments as in,^41^ we narrowed the search of relevant sequences to those likely to preserve the dehalogenation activity. Pfam MSAs are sometimes too broad, introducing large-gapped regions and making it difficult to design proteins with desired functions.^64^ To overcome this challenge, we used the EnzymeMiner web tool,^47^ which generates alignments specifically selected for function and catalytic site similarity (Figure 1A) as recently demonstrated on diverse enzyme families, such as fluorinases, marine bacterial flavozymes, and NADPH-dependent reductive aminases.^61,65−67^

The query of haloalkane dehalogenase (DhaA) from *Rhodococcus* strain TDTM0003 with UniProt ID P59336 yielded 22,567 sequences in EnzymeMiner. This extensive search resulted in the creation of a data set named HLDI-IV. To further refine the results, we preprocessed the resulting MSA against the DhaA query by removing protein sequences and positions with too many gaps. This step narrowed down the size of the alignment to 12,053 sequences and 299 positions, which were used for training. The HLDI-IV data set was utilized in both the first and second rounds of wet lab experiments (SI Section 2, Figure S1).

Based on the low experimental solubility observed in the first two rounds, we implemented a stricter protocol for creating the initial MSA. Inspired by Vasina et al.,^61^ we focused on more soluble HLD subfamilies I and II, generating a smaller MSA. This data set included the well-characterized DhaA enzyme from *Rhodococcus sp.* (UniProt ID P0A3G3), which served as the updated query for MSA preprocessing. We applied additional filters to reduce gap frequencies, lowering the threshold for gap column removal and filtering columns with frequent gaps, even if the query had an amino acid in that position, resulting in an MSA width of 293 positions with 4,053 sequences (HLDI-II dataset).

### Network Architecture Optimization

#### Variational Autoencoders Capture Sequence Spaces and Sequence Distribution

Replicating the methodology described by Ding et al.^41^ (SI Section 1.3), we demonstrated the capacity of variational autoencoders (VAEs) to delineate phylogenetic relationships among proteins in our HLDI-IV data set (Model 1). By encoding sequences into a latent space where evolutionary-related sequences map to nearby points, we observed a star-like configuration with multiple spikes radiating from a central point, reflecting the evolutionary divergence within the data set (Figure S3). This structure contrasts with the dispersed and unstructured representation of random sequences, highlighting that the latent space for our sequences captures their phylogenetic relationships, consistent with observations described in the original publication.^41^

**Figure 2:**
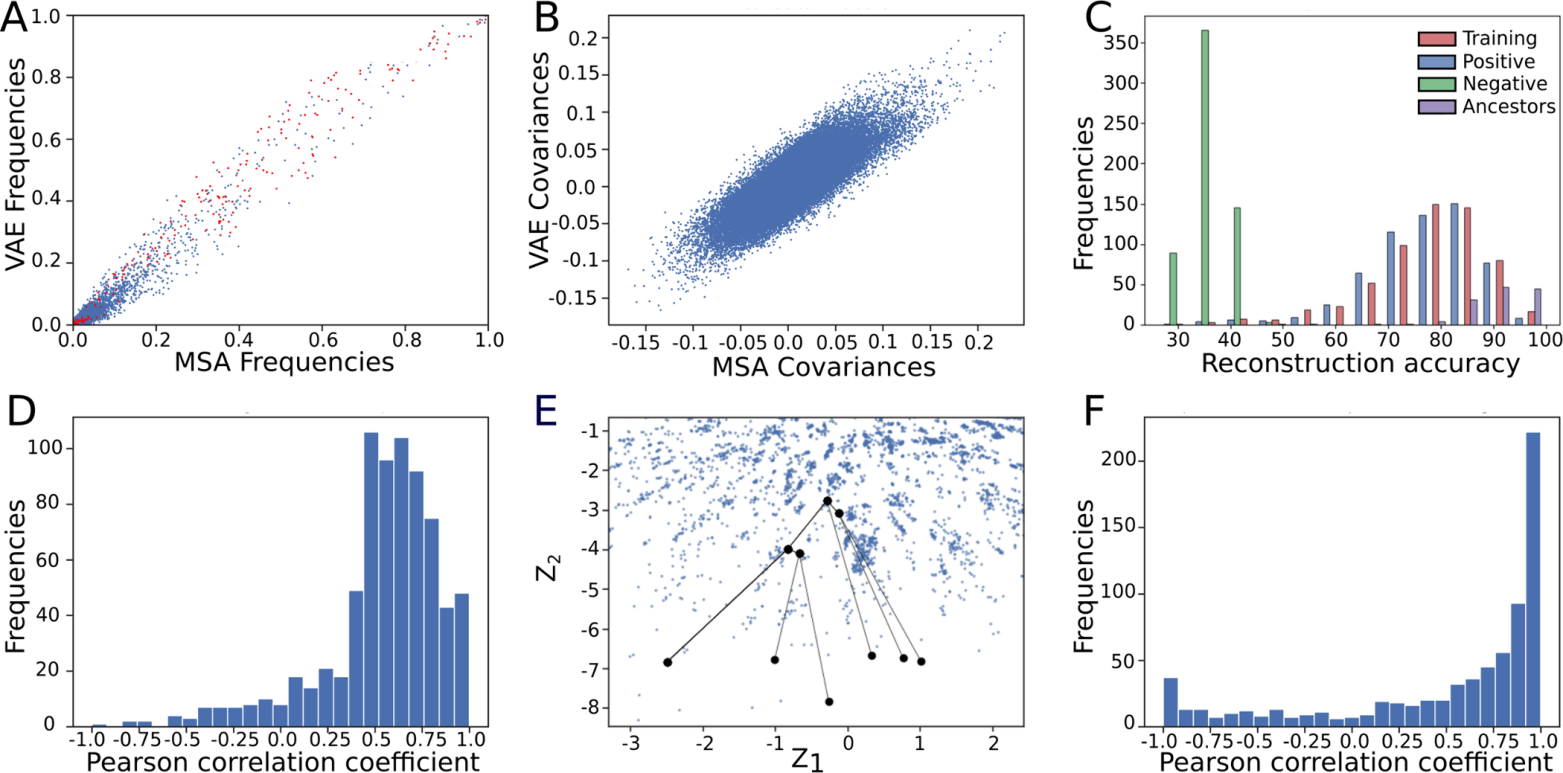
Showcases of the statistics used to measure the generative capacity of the final VAE model (Model 1, see SI Section 1) and the geometric properties of its latent space. (**A**) The first-order statistics for 3000 sequences randomly selected from the input MSA or VAE-generated. The red dots represent the gap symbol frequencies in sequence positions, while the blue points denote amino acids. (**B**) The second-order statistics demonstrate that our model can reconstruct pairwise amino acid occurrences fairly well (ρ = 0.68). (**C**) The average reconstruction accuracy for the negative (green), training (red), positive (blue), and ancestral (violet) control data sets. The shifts in the histograms between the sets imply that the model can distinguish random sequences (negative) from those in the input MSA (training and positive) and those corresponding to the straight-line strategy of generating ancestors (ancestors). (**D**) The Pearson’s correlation between depth in phylogenetic trees and latent space origin distance. Most sequences in tree branches have a positive correlation indicating that the latent space captures phylogeny. (**E**) Mapping a small phylogenetic tree onto the latent space. (**F**) Histogram illustrating the directional trends of phylogenetic tree branches projected onto the latent space. In this representation, 1 indicates a straight trajectory toward the latent space origin, while −1 represents the opposite trend. The histogram highlights that the majority of branches tend to align toward the latent space origin.

Before testing our hypothesis of generating ancestral-like sequences, we embarked on selecting the best model architecture based on the implementation provided by Ding et al.^41^ This involved optimizing the encoder, decoder, and training procedure to minimize the difference between the generated and input sequence distributions (generative capacity)^54^ while also preserving the relationship between phylogeny and the latent space (geometric properties). In order to evaluate the model’s generative capacity, we implemented several tests. The first test examined how well our model reproduces the statistics of the input data set on the output. To this end, we compared the first and second-order statistics of 3,000 randomly sampled MSA input sequences with those generated by our VAE model (Figure 2A-B) following the approach outlined in previous studies.^26,54,68^ The comparison revealed a close match between the two sets. We integrated query reconstruction accuracy^69^ as an additional metric in our analysis to ensure that the model was capable of reconstructing the query sequence with minimal mutations. Notably, the final Model 1 demonstrated the ability to reconstruct 98% of the query.

Our second test evaluated the model’s statistical profile by measuring the average reconstruction accuracy for sequences from various control sets. The test showed that the model could distinguish random sequences from MSA sequences using a reconstruction accuracy cutoff of around 50%. In other words, all the sequences from the negative set had an average reconstruction accuracy below this threshold together with only 23 of sequences from training and positive control sets, while the remaining 1201 sequences from these two control sets had an average reconstruction accuracy above this threshold (Figure 2C).

#### VAEs Capture Evolutionary Trends

To preserve evolutionary information in the latent space, we monitored the relationship between phylogeny and latent space geometry (Figure 2D-F). Phylogenetic trees with inferred ancestral sequences were mapped into the latent space, and we quantified the distance between latent space points and their corresponding positions in the phylogenetic tree (Figure 2D). Additionally, we analyzed the angle between vectors from leaf nodes to the origin and the first principal component of the branch’s latent coordinates (Figure 2F). Our results show that small dense encoder-decoder architectures capture evolutionary dependencies, while deeper architectures disrupt them (Figure S4). We set the dense layer width to match the protein sequence length and used a latent space dimensionality of 2 for simplicity and effective representation. Testing with higher-dimensional latent spaces did not yield significant improvements in reconstruction performance, further supporting our choice of a two-dimensional latent space (Table S1). Repeating this for Models 2–4 confirmed our findings with a correlation of 0.8, supporting our choices for layer width and latent dimensionality (SI Section 2).

### Construction of the Evolutionary Trajectory

#### The Latent Space Captures Protein Stability

**Figure 3:**
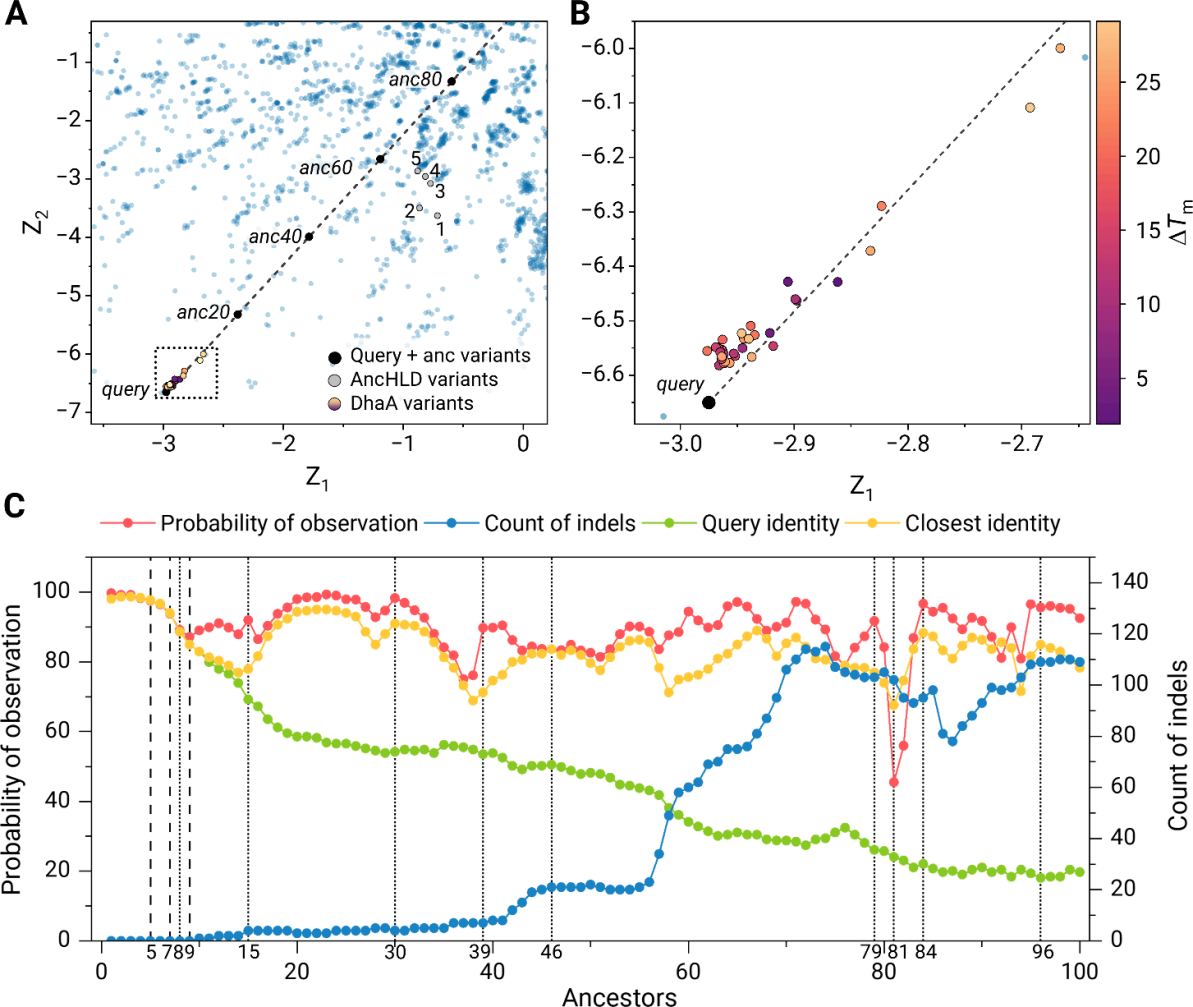
The straight-line evolutionary strategy for Model 1. (**A**) Straight-line evolutionary strategy reconstructed 100 sequences along the trajectory from query embedding to the latent space origin (black dashed line). The embeddings of previously characterized ancestors (gray points 1–5 denoting AncHLD variants of the respective number^48^) and engineered DhaA variants^49^ (magma spectrum points) are mapped closer to the latent space origin, supporting the idea behind our ancestral generation strategy. (**B**) A detailed view of the previously engineered DhaA variants. While there is no strong correlation between the positions in the latent space and the stability gain (Δ*T*_m_) of variants up to 28 °C, some of the most stable points are situated closer to the origin. (**C**) The statistical profile of 100 sequences from the straight-line evolutionary strategy. The vertical lines represent sequences selected for experimental characterization for the first and second rounds (Table S2) where dashed line variants were successfully expressed, while for dotted lines, no soluble expression was observed. The ancestors are numbered 1 to 100 based on their order in the VAE-generated latent space, with lower numbers being closer to the starting sequence and higher numbers representing more divergent designs closer to the latent space origin. Number 0/Query represents the reconstruction of the original embedding of the query sequence.

The ancestral sequences are often associated with enhanced stability compared to their extant counterparts.^23,70^ We hypothesized that the structure of the latent space might encode stability and place more stable variants of our target protein DhaA closer to the origin compared to the wild type. To test this hypothesis, we mapped two sets of experimentally measured stability values to the latent space of Model 1. The first set consisted of six ancestral sequences from the previous protein engineering campaign (DbjA, DbeA, DhaA, DmxA, and DmmA).^48^ The second set consisted of 24 previously engineered DhaA variants based on the FireProt method.^22,49,50^ Both data sets had latent space coordinates closer to the origin of the latent space (Figure 3A-B), supporting the notion that the latent space captures the information about stability. The observations were also recapitulated for HLDI–II data set and Model 2.

#### Latent Space Strategy Guides the Search and Selection of Sequences

The regular distribution of stable sequences in the latent space (Figure 3B) led us to develop the *straight-line evolutionary strategy*. This strategy encodes the query sequence (DhaA in our case) into its latent representation and then follows the straight line connecting that point to the origin of the latent space (Figure 3A), mimicking the mapping of ancestral dependencies into the latent space. In our experiments, the line is divided into 100 equal intervals, whose boundaries are then selected for reconstruction by the decoder. The motivation for the *straight-line evolutionary strategy* is based on the observation by Ding et al.^41^ that ancestors tend to be placed closer to the origin of the latent space. Therefore, sequences reconstructed in this direction from the latent space might be ancestor-like, e.g., show increased stability. For the sake of brevity, in what follows we will refer to these ancestor-like designs generated by the decoder from the straight-line evolutionary strategy in the latent space as “ancestors” and use the prefix “Anc”.

To represent the designed sequences, we analyzed several statistical parameters for the individual designed sequences: the average reconstruction probability, similarity to the query sequence, similarity to the closest sequence from the training set, and the number of insertions/deletions compared to the query sequence. The values obtained were plotted and visually inspected to identify variants with interesting statistical values. The generated profiles were then used to select suitable variants for subsequent experimental characterization (Figure 3C).

Using the statistical profile, we identified 9 promising designs in the first round for further laboratory experiments to gain deeper insight into the statistical indicators. These designs exhibited a wide range of sequence variability, ranging from 45 substitutions and no insertions or deletions (indels) to 138 substitutions and 109 indels (Table S2) (AncDhaA1–9). The substitutions and indels, with deletions in most cases, covered the entire protein structure. In the second round, we focused on the more conserved variants (ancestors 5, 7, and 8 in Figure 3C; AncDhaA10–12 for reference in experiments) with 7 to 34 substitutions without indels. Altogether, 12 designs were selected for laboratory expression and biophysical characterization from Model 1.

### Variant Selection Conditioned for Soluble Proteins

#### Solubilization of Designs by Introducing New Knowledge to the Data Set

As we observed low solubility in the first round (see Section Experimental Characterization of Expressed Variants and Figure S10A-B), we incorporated previous findings on the low solubility of HLD subfamilies III and IV^61^ in our workflow. To this end, we embarked on a third round of experiments restricted to the HLD subfamilies I and II. This round focused on training additional models (Models 2–3) and designed eight DhaA variants (AncDhaA13–20, Table S2). The first candidate VAE (Model 2) achieved up to 97% similarity in query sequence reconstruction with satisfactory second-order statistics. Considering the often-disruptive impact of indels, we selected and curated five designs from the straight-line evolutionary strategy accumulating at most one indel (AncDhaA13–17). Another VAE was trained from a different initialization (Model 3). During analysis of this model, we found that at the end of reconstructed sequences, it incorporated a His-tag sequence, common peptide tag for protein purification. The inclusion of the His-tag likely happened during the training phase, which influenced the model’s generation of sequences. Overall, 42 out of 100 reconstructed sequences from the *straight-line evolutionary strategy* exhibited the closest sequence similarity toward 5 different protein sequences of PDB structures found in HLDI–II data set. Additionally, Model 3 had a curious pattern for the origin of the latent space: the model demonstrated a significant shift in sequence similarity toward DbjA. Therefore, we selected ancestor 99 (AncDhaA20) from this model for further experimental characterization to explore its unique shared sequence similarity to both DhaA (52%) and DbjA (93%).

#### Solubilization of Designs by Conditional Variational Autoencoder

Finally, in the third round, we also decided to explore one more solution to low solubility, conditional variational autoencoders (CVAEs). To this end we added solubility scores from SoluProt^52^ to the training, discretized into three bins for low, moderate, and high solubility values (Model 4) (Figure S6A). We conditioned the sampling process from Model 4 using a straight-line evolutionary strategy on the highest bin label forcing CVAEs to introduce patterns from sequences with predicted high solubility in decoded designs (Figure S5). We took two variants from Model 4: ancestor 0 (AncDhaA18) with 30 mutations as the control of the pattern extracted from highly soluble sequences and ancestor 18 (AncDhaA19), which had 49 mutations and one deletion in the coil region at position 32 (Figure S6B), as it exhibited high confidence in the model (reconstruction probability of 93.28%) and an increased number of high probable residues (85 positions scored above 90%).

#### Refining the Mutations by Manual Curation of the Structures and Stability Scores

From Model 2, we selected three ancestors with unique features: ancestor 3 closely mimicked the wild type with 98% similarity; ancestor 16 notably introduced a proline at position 75; and ancestor 23 was the last variant starting with a regular sequence pattern (MSEIGT), suggesting high solubility and expression potential based on its 88% similarity to the PDB sequence4WCV. To increase our chances of producing soluble variants, we manually curated proposed mutations based on the structural predictions from AlphaFold^16^ and stability assessments using the MutCompute tool^57^ (Table S3). Mutation manual curation led to the classification of VAE-proposed mutations into safe and risky categories, respectively (SI Section 3). Starting from ancestor 3, we kept four nonrisky mutations. We removed two structurally and statistically risky mutations P34V and L238F from the original six-point variant proposed by the VAE, producing the design AncDhaA14. As experimental validation of identified risky mutations, we selected ancestor 0 with eight substitutions (AncDhaA13). For the second manually curated variant (AncDhaA16), we selected ancestor 15 as the template, which included as the last VAEs ancestor with no insertions, and we removed the risky mutations P34V and L238F, leaving nine mutations compared to the DhaA wild type. To experimentally determine the effect of suggested proline insertion with risky mutations, we selected ancestor 16 as predicted by VAEs (AncDhaA15) (Figure S2). As a template for the third manual curation target, we selected ancestor 23. We incorporated all mutations suggested by the VAEs except the risky ones (P34V, L238F) and the proline insertions, resulting in a final design carrying 32 mutations (AncDhaA17).

#### Investigation of Trajectory Mutational Patterns in VAEs Designs

To gain a comprehensive understanding of how VAEs propose mutations across designs, we further analyzed mutational patterns along the evolutionary trajectory generated by VAEs Model 2. The analysis indicated that mutations are not distributed randomly but tend to accumulate at specific positions, suggesting a targeted evolutionary trajectory rather than a stochastic process. Additionally, certain mutations introduced in earlier designs were retained in subsequent designs, while others were reverted, indicating an iterative refinement process. Further details on the mutational profiles and alignment patterns of the generated ancestors are provided in SI Section 6 and Figure S8, S9.

### Experimental Characterization of Expressed Variants

#### Variant Production

Protein overexpression, purification and assessment of solubility and whole-cell activity testing was carried out in three rounds (Table S2, SI Section 7), yielding 9 soluble variants: AncDhaA1 (round 1), AncDhaA10–11 (round 2) and AncDhaA13–16, 18, and 20 (round 3). Thus, the success rate in obtaining soluble variants gradually increased from 11% in round 1 to 67% in round 2 and reaching 75% in round 3. The whole-cell activity screening by HOX assay revealed that 6/9 soluble variants were active with a benchmark substrate 1,2-dibromoethane (Figure S11).

#### Secondary Structure Analysis

**Figure 4:**
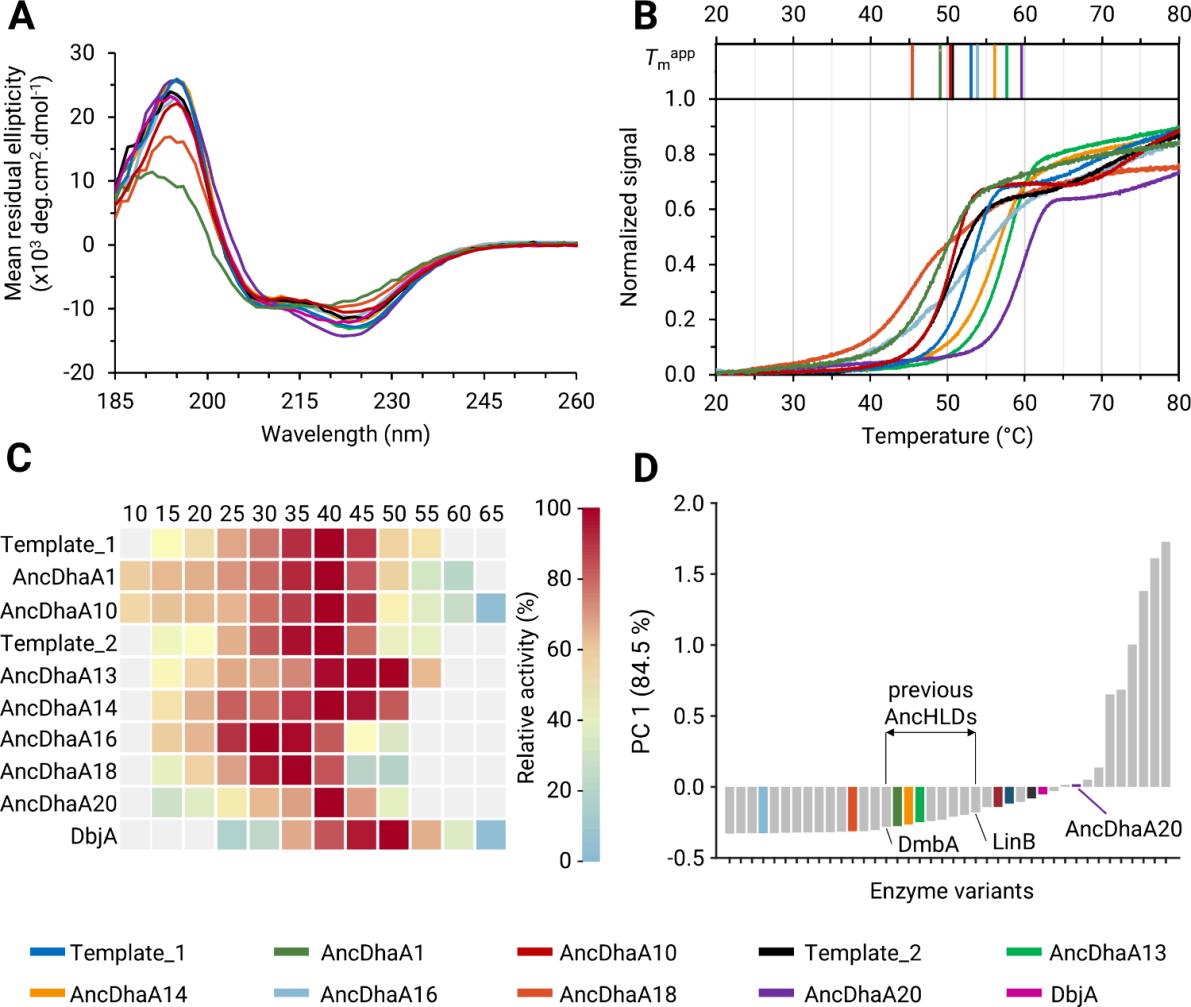
Experimental characterization of selected variants. (**A**) Far-UV circular dichroism spectra probing the correct folding and secondary structure of the variants. (**B**) Normalized thermal denaturation curves from nanoDSF spectroscopy with apparent melting temperatures (*T*_m_^app^) are shown above the curves. (**C**) The dependence of specific activity on temperature. The heatmap represents the relative activity of individual variants. (**D**) The score plot shows the first principal component PC 1 explaining 84.9% of the data variance, which compares VAE-based designs (in color) with previously characterized wild-type haloalkane dehalogenases (gray)^61^ in terms of their activity with 27 substrates being determined by the MicroPEX method.^62^ The highlighted range between DmbA and LinB corresponds to the ranges of values observed for previously characterized AncHLD variants.^48^ The values of PC1 above this range imply that the overall activity of the corresponding designs was higher than those for previous AncHLD variants. The color code for individual variants is shown at the bottom.

To confirm the proper folding of the studied variants, circular dichroism (CD) spectra were collected for all soluble variants (Figure 4A). Overall, CD spectra of most variants highly resemble those of the templates (typical α/β-hydrolase fold), confirming proper folding. On the contrary, the spectra of AncDhaA1, AncDhaA11, AncDhaA15, and AncDhaA18 (Figure S12) deviated from the templates. To further understand the secondary structure of the variants, BeStSel server^60^ was used for fitting experimental data and analysis of PDB structures, and PDBMD2CD^59^ was used for predicting CD spectra from experimental structures of templates and AlphaFold models of selected variants. Figure S12B shows that the prediction of CD spectra based on AlphaFold structures did not match the experiments in all the variants. This highlights a limitation in AlphaFold’s ability to accurately predict changes in folding and emphasizes the need for further improvements in computational methods. Experimental validation remains essential to address this challenge.

#### Thermostability

Thermodynamic stability of all variants was assessed by nanodifferential scanning fluorimetry (Figure 2B, Table S4). The apparent melting temperatures for the variants were in the range of 45 °C–60 °C. AncDhaA13, AncDhaA14, AncDhaA16 and AncDhaA20 surpassed the respective query in terms of apparent melting temperature. The highest Δ*T*_m_ of 9 °C was measured for AncDhaA20. Protein aggregation was observed for the variants AncDhaA1 and AncDhaA20, showing the onset at 45.5 and 48.5 °C, respectively (Figure S13).

#### Temperature Profiles

We then proceeded to measure temperature profiles (Figure 4C). Most variants showed the *T*_max_ (temperature at which maximum activity was detected) of 40 °C, which is in agreement with previously determined temperature profiles for DhaA.^62^ Notably, AncDhaA13 showed *T*_max_ of 50 °C, which aligns with its increased thermostability. The temperature profiles for AncDhaA11 and AncDhaA16 were not obtained, as the activities were below the detection limit. Due to compromised activity and folding, both variants were excluded from the subsequent substrate specificity profiling.

#### Substrate Specificity

The temperature of 35 °C was selected for the subsequent specificity characterization for being below the onset of denaturation (Table S4) for most of the remaining variants and close to their *T*_max_ values. To explore the obtained substrate specificities in the context of the haloalkane dehalogenase family, the principal component analysis (PCA) was conducted by augmenting the previously used data set comprising substrate specificities for 32 wild-type dehalogenases^61^ with the newly obtained data. The PCA of raw data (Figure 4D) as a standard representation of overall dehalogenase activity^46,61,71^ showed that AncDhaA20 surpassed both Templates, DbjA and the previously characterized AncHLD variants.^48^ The higher values of PC1 indicate the higher overall activity, as the first principal component corresponds to the weighted average of all the activities, shifted to be centered around zero. The acquired substrate specificity data for previously characterized AncHLD variants are not directly comparable with the MicroPEX data, however, due to different assays used. Therefore, only an approximate area of graph where AncHLD variants stand in terms of overall activity could be determined (Figure 4D). The overall activity of AncDhaA10 was also higher than the previously characterized AncHLD variants, on the level of the wild type. Three more variants showed activity in the ranges of previously characterized AncHLD, and two more below (Figure S14).

The analysis of log-transformed activity data (Figure S15) further showed that AncDhaA1, AncDhaA10, AncDhaA13, and AncDhaA14 differed only very slightly from templates in their substrate preferences. The profiles of AncDhaA18 and AncDhaA20 resembled more closely the specificity profile of DbjA, which is not unexpected in the case of AncDhaA20 due to its high sequence similarity. AncDhaA16 differed significantly from all other variants due to the low number of converted substrates (10 out of 27). Probable activity with other substrates could have ended below the limit of detection (different for each substrate, in the range of 10–100 μM).

## Discussion

In this study, we utilized variational autoencoders (VAEs) to map functionally related haloalkane dehalogenase sequences from EnzymeMiner onto VAEs latent spaces, following an approach inspired by Ding et al.^41^ This process revealed that the latent space could capture the phylogenetic relationships of the sequences, which motivated us to employ the VAE framework to create ancestral-like variants of the haloalkane dehalogenase DhaA. We tuned the network hyperparameters to accurately reflect the statistical frequencies of the input while maintaining the relationship between evolutionary trajectories and latent spaces. We discovered that a simple feed-forward neural network with a single dense layer matched to the input MSA columns and the two-dimensional latent space^42^ was enough for this task.

We then introduced a simple strategy to generate novel sequences based on the geometry of the latent space. We achieved this by reconstructing the embeddings along the trajectory toward the origin of the latent space. Employing this strategy, we systematically executed the pipeline in three rounds of laboratory experiments and optimization, leading to four VAEs models producing 20 designs in total. Similarly to the study of computational filter evaluation for synthetical protein designs from generative models,^72^ each iteration revealed new insights, allowing for iterative refinement and improvement of our approach. Notably, we increased the success rate of soluble designs from 11% in the first round and 66% in the second round to 75% in the third round, illustrating the effectiveness of applying accumulated knowledge for improvement (SI Section 2).

In the first two rounds, we faced solubility issues, with three designs (AncDhaA1, AncDhaA10, and AncDhaA11) showing sufficient expression for detailed characterization (secondary structure, thermostability, temperature profiles, and substrate specificity) including the in-house microfluidic device MicroPEX^73^ . Surprisingly, the predicted CD spectra from AlphaFold structures differed significantly from experimental data (Figure S12). While AlphaFold predicted native-like structures, experiments revealed misfolding, suggesting a bias toward native folds in AlphaFold predictions for synthetic sequences generated by protein language models.^74^ Thermostability analysis showed no significant changes, and AncDhaA10 exhibited above-average activity among the haloalkane dehalogenases.

In the third round, we addressed solubility by refining the input MSA to HLD subfamilies I–II, applying stricter preprocessing to suppress indels, and manually curating sequences by incorporating AlphaFold^16^ and MutCompute^57^ stability assessments. These steps improved solubility in most designs. Interestingly, the noncurated AncDhaA13 showed good solubility and activity despite risky mutations (P34V and L238F), highlighting the limits of current tools in predicting epistasis effects. On the other hand, manual curation rescued poorly soluble AncDhaA17, which informed the design of AncDhaA16 and predicted the disruptive impact of a proline insertion on AncDhaA15’s activity. We also observed that even despite a large number of mutations (51.4–98.5% sequence identity to template, Table S4), all soluble designs showed stability at least comparable to that of the WT (the smallest *T*_m_^app^ was 45.4 °C), which further emphasizes the utility of VAEs in suggesting new protein sequences. Furthermore, in terms of overall activity, 5 out of 7 variants showed comparable or higher activity than previously characterized AncHLD variants^48^ (Figure 4D).

A different initialization of a model training and stricter threshold of column removal with query amino acid positions in the third round led to a second VAE model, which generated sequences with an implicit His-tag and high similarity to proteins with known experimental structures. Investigating the sequence reconstructed from the origin of the latent space (AncDhaA20) unveiled a notable shift in similarity toward another wild-type dehalogenase, DbjA,^75^ altered substrate specificity, increased thermostability (60 °C) and improved activity (3.5-fold), being a top-performing haloalkane dehalogenase (Figure 4D, Table S4). To better understand the sensitivity of the *straight-line evolutionary strategy* to different initializations, we examined the embeddings over an ensemble of four randomly initialized VAEs (SI Section 5, Figure S7). Although we observed a general trend that the straight-line evolutionary trajectories converged toward the origin of the latent spaces of different VAEs, it was also evident that the trajectories exhibited quite wide scatter. This suggests that ensemble learning^76^ might be an interesting direction for follow-up research to improve the robustness of our strategy.

Another promising direction for further improvement include developing better scoring methods for the sequences generated by protein language models, particularly those that will allow filtering out misfolded or poorly soluble designs *in silico*, and adopting the recent developments in transformer-based architectures, which have demonstrated a better capacity for learning from amino acid sequences.^77,78^ Integrating transformer-based architectures with manifold learning can further enhance their ability to generate sequences of stable and soluble proteins.^42,43^ To bolster the robustness of future studies, adopting a generation protocol for ancestral sequences that incorporates an ensemble of models might also be advantageous.^79^ This approach addresses the observed instability of ancestral trajectories within the latent space and could establish a more reliable foundation for future investigations.

## Conclusions

Our study demonstrated that the structure of the latent space and the generative potential of VAEs are capable of guiding the sequence search and designing novel soluble and functional proteins with enhanced stability. The workflow underwent systematic improvements through three consecutive design-build-test phases, with each iteration informed by the findings from the previous one. The success rate of soluble designs increased from 11% in the first round to 66% in the second round and 75% in the third round. Through this process, complemented by manual curation of specific variants, we achieve a notable increase in stability—up to 9 °C for the top-performing AncDhaA20 variant, with an average improvement of 3 °C and a significant boost in activity up to 3.5-fold. The frequency and location of indels were the most critical parameters. In general, we recommend selecting designs with a low number of indels or with high protein similarity to natural sequences, preferably to those in PDB. A current limitation of our study is that it was conducted using a single enzyme family. Validation of designs from other protein families will help understand the generalizability of the developed approach. Overall, our study demonstrates that VAEs represent a promising strategy for generating novel soluble, stable, and functional enzymes.

## Supporting Information Available

Additional *in silico* and wet lab experimental details, materials, and methods, including the workflow overview, list of protein designs, analysis of introduced mutations, and detailed experimental characterization (PDF)
