# Supplementary material for: Engineering Dehalogenase Enzymes Using Variational Autoencoder-Generated Latent Spaces and Microfluidics
Source: JACS Au. 2025 Feb 13;5(2):838–50. doi: 10.1021/jacsau.4c01101 (PMC11862945; doi:10.1021/jacsau.4c01101)
Supplement: Supplementary file 1 — au4c01101_si_001.pdf [file au4c01101_si_001.pdf]

Supporting Information

Engineering Dehalogenase Enzymes using Variational Autoencoder-Generated Latent Spaces and Microfluidics

Pavel Kohout[#,a,b], Michal Vasina[#,a,b], Marika Majerova[a,b], Veronika Novakova[a,b], Jiri Damborsky[a,b], David Bednar[a,b], Martin Marek[a,b], Zbynek Prokop[a,b,*], Stanislav Mazurenko[a,b,*]

[a] Loschmidt Laboratories, Department of Experimental Biology and RECETOX, Faculty of Science, Masaryk University, Brno 611 37, Czech Republic

[b] International Clinical Research Centre, St. Anne's Hospital, Brno 656 91, Czech Republic

[#] These authors contributed equally.

[*] Corresponding authors: Stanislav Mazurenko - mazurenko@mail.muni.cz; Zbynek Prokop - zbynek@chemi.muni.cz.

1. Data acquisition and model evaluation

1.1. MSA and data preprocessing

The HLDI-IV dataset was created by the EnzymeMiner tool [1]. As an input sequence, 3.8.1.5 - Haloalkane dehalogenase was used, and from the sequence selection table, all 33 provided sequences together with their essential residues were selected. In advanced options, only the maximum number of hits in PSI-BLAST was changed to 50,000. The resulting dataset was composed of 22,567 sequences (job ID xvmwa7). For a given query sequence, the MSA was preprocessed through several steps: (i) the gap positions in the query were removed for every input sequence except for the columns where fewer than 20% of sequences had gaps, as these positions are considered evolutionarily important and retaining them enables the incorporation of informative insertions in the designs; (ii) sequences with gaps in more than 40% of positions were removed; (iii) sequences were clustered by 90% identity to support the diversity, and only one sequence for each cluster was picked for the training dataset, together with the query sequence, (iv) sequences with less than 50% overlap with the query were excluded. Similar to [2], each sequence in the MSA was then represented as a matrix of the size L x 21 with one-hot encoded residues, where L stands for the number of positions in the final alignment (299), and 21 columns correspond to 20 amino acids and a gap. The sequence P59336_S14 of DhaA (Haloalkane dehalogenase from *Rhodococcus sp*) was chosen as a query for the training process. This resulted in 12053 sequences with 299 positions in the MSA left for the training after the preprocessing.

The HLDI-II dataset for training models used in the third round of experiments was created by a similar protocol. As input sequences, only DhlA and LinB were selected, together with all 19 sequences in the "Other known sequences" input field. The resulting dataset was composed of 4,053 sequences (job ID d6cv1o). As a query for MSA preprocessing, the DhaA sequence (Uniprot ID P0A3G3) was selected. Experimental results from rounds 1 and 2 indicated that the poor solubility might stem from many deletions suggested in the reconstructed sequences. Therefore, we adjusted parameters of MSA preprocessing to lower the number of gaps. In addition to the previous preprocessing steps, we implemented a further refinement by excluding columns from the MSA where amino acid symbols were present in the query if the column contained more than 80% gaps (>70% for Model 3). These excluded query amino acids were stored separately and reintegrated into their original positions in the generated sequence. Also, we did not perform 90% identity sequence clustering.

1.2. Model generative capacity

The first-order statistic is a comparison of the frequency of occurrence of each amino acid on each position between two sets of data. The frequency of amino acid α at position *i* in MSA with *N* sequences is given by the following formula:

$$f_{\alpha}^{i} = \frac{count_i(\alpha)}{N}, \qquad \textbf{(Eq. 1)}$$

where $count_i(\alpha)$ is the number of occurrences of the amino acid α in the MSA. The second-order statistics are similar but for two positions i, j:

$$f_{\alpha\beta}^{ij} = \frac{count_{i,j}(\alpha,\beta)}{N}, \qquad \textbf{(Eq. 2)}$$

where $count_{i,j}(\alpha,\beta)$ is the number of occurrences of the amino acid pair (α,β) in the MSA. To gain more insight into the distribution of features in the generated dataset, we further computed the pairwise covariance score given by the following formula:

$$C_{\alpha\beta}^{ij} = f_{\alpha\beta}^{ij} - f_{\alpha}^{i} f_{\beta}^{b}, \qquad \textbf{(Eq. 3)}$$

where $f^{ij}{}_{\alpha\beta}$ and $f^{i}{}_{a}$, $f\ ^{b}{}_{\beta}$ were the second and first-order statistics for columns i, j of the alignment, respectively. Each covariance term measures the difference between the joint frequency for pairs of amino acids and the product of the frequencies of the residues at each site, i.e. the expected counts under the statistical hypothesis of independence. Therefore, zero $C^{ij}{}_{\alpha\beta}$ for all $\alpha\beta$ would imply frequences one would observe if positions i, j were independent. Reproducing pairwise covariance in protein alignments is an important aspect of generative models as it measures how well the model captures interactions between distant amino acids, an essential indicator for the likely stability and function of the generated proteins [3].

For each model, we compared the pairwise covariance scores for all positions and residuals in generated ($\tilde{C}^{ij}{}_{\alpha\beta}$) and the input ($C^{ij}{}_{\alpha\beta}$) alignment using the Pearson correlation coefficient $\rho(\{C^{ij}{}_{\alpha\beta}\},\{\tilde{C}^{ij}{}_{\alpha\beta}\})$. In the case of this study, the statistics were calculated for the input and synthetically generated datasets having 3,000 randomly selected samples. Synthetic data were reconstructed from the latent space points sampled according to the a priori chosen distribution, i.e., a Gaussian distribution with the zero mean and the variance of 2.

1.3. Phylogeny mapping and evaluation

We generated 13 phylogenetic trees using our input MSA dataset for the latent space phylogenetic analysis. Each tree consisted of approximately 100 randomly sampled leaf nodes from the preprocessed MSA. Using our fully automated in-house ancestral sequence reconstruction tool FireProtASR [4], we obtained an average of 10 levels in each phylogenetic tree. To explore the relationship between the tree branches and the latent space, we mapped each individual branch, along with its reconstructed ancestral sequences, into the latent space. We then measured the correlation between the depth of a node (i.e., the distance between the root and the node) and the distance of the corresponding ancestral node's latent embedding from the origin of the latent space, following an approach similar to [2]. In addition, we sought to gain insights into the reconstruction strategy by evaluating the direction in which the tree branches were mapped in the latent space. Specifically, we calculated the vector representing the first principal component of each branch and computed the dot product with the vector pointing from the embedding of the leaf node sequence to the origin of the latent space. This analysis allowed us to compare the trajectory directions of the different tree branches, and we reasoned that the straight evolutionary strategy would generate ancestral-like sequences.

1.4. Architecture selection

The model architecture consists of several components: an encoder, decoder, latent space, and a loss function, each with parameters affecting its generative performance. These components are crucial in reconstructing protein, replicating order statistics of the original dataset, and capturing phylogenetic information in the latent space, which is a key focus of this study.

We tested the application of multiple fully dense layers in both the encoder and decoder structures, aiming for improved generative performance (**Table S1**). However, this approach compromised the phylogeny-informed structure encoded in the latent space. We found that a single dense layer effectively captures phylogenetic relationships while scaling appropriately to the preprocessed MSA width. This single dense layer can reconstruct query sequences with as few as three mutations, demonstrating reasonably high generative performance. We also tested a convolutional neural network as encoder and decoder, but this change significantly weakened the relationship between phylogeny and the geometry of the latent space.

We reduced the contribution of the regularization term in the loss function to prioritize reconstruction error, but we did not observe a significant impact on generative performance. Lowering

the weight decay factor from the original value of 0.1 to 0.0 significantly improved reconstruction accuracy while maintaining the phylogenetic structure in the latent space. It is important to note that a weight factor of 0.0 results in a latent space structure that is less clustered, making the star-like shape less pronounced but still preserved. Additionally, we implemented a linear decrease of the weight decay factor over the first quarter of the training epochs, reducing it from 0.1 to 0.0. We found this approach to be a good compromise, allowing for a relaxation of the latent space structure while achieving desirable reconstruction outcomes used in the third round of experiments.

After completing the final fine-tuning, the model with a decay weight factor set to 0.0 and a decreasing decay factor during the first quarter of the epochs in the third round exhibited the best performance. This model, which includes one hidden layer with several neurons equal to the width of the MSA, was selected as the base model for further evaluation and ancestral sequence generation. The final training protocol is available at https://github.com/loschmidt/vae-dehalogenases.

Table S1: The effect of latent space dimensionality on the model reconstruction accuracy. Several parameter tests were conducted while holding the weight decay factor at 0.0, using 100 neurons in a single hidden layer and applying early stopping. The table presents the performance of different latent space dimensionalities in reconstructing the query DhaA score and the pairwise reconstruction accuracy for sequences from the HLDI-IV dataset. The results indicate a slow improvement with increasing latent space dimensionality. However, higher dimensionalities also sometimes exhibited collapsed dimensions [5], where some dimensions did not follow a normal distribution (e.g., at 33 dimensions). We opted for a simple and interpretable two-dimensional latent space representation based on these findings.

Dimensions	Pairwise reconstruction (%)	Query reconstruction (%)
2	73.16	90.06
3	74.97	79.38
4	75.80	84.06
5	76.51	79.37
6	76.75	85.31
8	77.57	76.56
16	79.00	94.06
20	79.33	92.50
33	80.23	60.63

2. Workflow overview

This section aims to present a comprehensive overview of the experimental sequence undertaken and the individual designs derived from it. The project timeline consists of the iterative exploration of multiple rounds of experiments, models, and datasets to leverage our cumulative online learning experience. For clarity and visualization, we have included **Fig. S1**, which delineates the timeline of experiments alongside a detailed list of the generated designs. This visual aid provides a structured representation of the temporal progression of our experimental efforts and the resulting designs.

Initially, we trained Model 1 on the HLDI-IV dataset. Subsequently, employing an evolutionary trajectory approach described in the main text, we generated 100 VAE-based ancestors starting from the sequence DhaA from *Rhodococcus sp*. TDTM0003, named as Template 1 (UniProt P59336, designated P59336_S14 within the HLDI-IV dataset). From these ancestors, we identified nine variants based on various profile statistical values, delving deep into the latent space center. Only the first variant, ancestor 9, was expressed in sufficient concentrations and showed HLD activity during the first round of experiments (**Fig. S1**). Consequently, we proceeded to the second round of experiments, adopting a more conservative approach by focusing on three variants closer to the query embeddings within the same evolutionary trajectory.

The previous study by Vasina et al. [6] highlighted the low solubility of members within the HLD subfamilies III and IV. Consequently, we initiated a third round of experiments to train additional models using the HLDI-II dataset only. This dataset includes the well-characterized DhaA enzyme from *Rhodococcus sp.* (UniProt P0A3G3, Template 2, designated as DhaA_S19 in the HLDs I-II dataset), serving as the reference for all models in this round. Model 2 demonstrated a high degree of query sequence reconstruction, achieving up to 97% similarity and satisfactory second-order statistics (**Fig. 2** and **S4**). We selected five designs out of 100 ancestors from the evolutionary trajectory for further exploration, some of which underwent manual refinement (see **SI Section 3** for details).

Additionally, we adjusted our approach to MSA preprocessing by implementing column filtering thresholds to reduce the number of insertions/deletions in the proposed designs, identifying these as a primary cause of design failures. Specifically, we excluded any columns from the MSA that contained gaps in more than 30% of occurrences. This preprocessing step led to the development of Model 3, which exhibited superior reconstruction capabilities and second-order statistics of 99% and 80%, respectively. Interestingly, despite removing only one additional column, resulting in a 290-column wide multiple sequence alignment, Model 3 succeeded in designing sequences with a high resemblance to known PDB sequences within the HLD I-II dataset, and it even autonomously added a His-tag at the end of the design. Moreover, Model 3's ancestor 99 (AncDhaA20) showed a notable shift in sequence similarity towards DbjA (93% and 52% sequence identity to DbjA and the query, respectively), prompting further investigation due to its potential unique properties, such as altered substrate specificity and enhanced stability, given its near-central position in the latent space.

Finally, we examined the potential of a conditional variational autoencoder (Model 4) to generate sequences based on SoluProt solubility scores [7], selecting two designs (AncDhaA18-19) for their promising attributes.

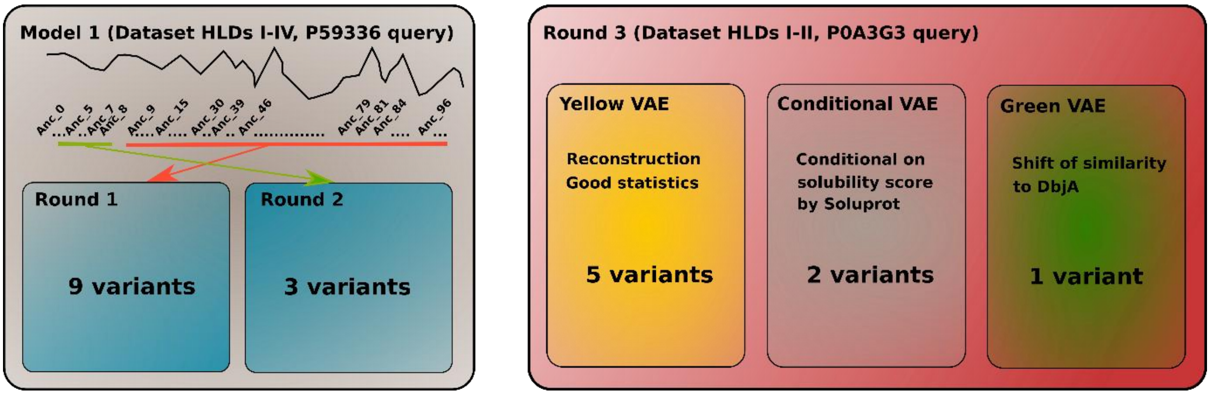

Fig. S1: **The overview of experimental rounds, VAE models and datasets used in this study.** We implemented three design-validation rounds within our study. We used designs generated from Model 1 for the first two rounds and only trained on the HLDI-IV dataset. In the third round with refined dataset HLDI-II, we trained three models different in MSA preprocessing (with or without stricter cutoffs for gaps) or method (classical or conditional VAE) and selected eight variants.

Table S2: Overview of characterized DhaA ancestors by laboratory experiments. Query Template 1 DhaA wild type (Uniprot ID P59336) is composed of 294 amino acids. Template 2 is the query used in Model 2, Model 3, and Model 4 (Uniprot ID P0A3G3). The ancestor column indicates ancestor order in the evolutionary trajectory (Ancestor 0 is the reconstruction of the wild type, and Ancestor 100 is in the origin). Identity to closest column specifies the sequence similarity to the closest sequence in the dataset. Sequences marked with an asterisk underwent manual curation.

Design	Ancestor	Mutations vs. template	identity to closest (%)	Round	Model	Expressed?
Template 1	query - P59336	0 substitutions, 0 indels	100	1,2	1	Yes
AncDhaA10	Ancestor 5	7 substitutions, 0 indels	97.6	2	1	Yes
AncDhaA11	Ancestor 7	18 substitutions, 0 indels	93.7	2	1	Yes
AncDhaA12	Ancestor 8	34 substitutions, 0 indels	88.6	2	1	No
AncDhaA1	Ancestor 9	45 substitutions, 0 indels	84.9	1	1	Yes
AncDhaA2	Ancestor 15	88 substitution, 4 indels	77.9	1	1	No
AncDhaA3	Ancestor 30	133 substitution, 4 indels	90.9	1	1	No
AncDhaA4	Ancestor 39	132 substitution, 7 indels	71.2	1	1	No
AncDhaA5	Ancestor 46	127 substitution, 21 indels	83.6	1	1	No
AncDhaA6	Ancestor 79	118 substitution, 103 indels	76.9	1	1	No
AncDhaA7	Ancestor 81	125 substitution, 102 indels	67.5	1	1	No
AncDhaA8	Ancestor 84	138 substitution, 95 indels	88.2	1	1	No
AncDhaA9	Ancestor 96	136 substitution, 109 indels	84.9	1	1	No
Template 2	query - P0A3G3	0 substitutions, 0 indels	100	3	2,3,4	Yes
AncDhaA13	Ancestor 0	8 substitutions, 0 indels	97.2	3	2	Yes
AncDhaA14*	Ancestor 3	4 substitutions, 0 indels	98.6	3	2	Yes
AncDhaA15	Ancestor 16	12 substitutions, 1 insertion	95.5	3	2	Yes
AncDhaA16*	Ancestor 15	9 mutations, 0 indels	96.9	3	2	Yes
AncDhaA17*	Ancestor 23	32 mutations, 0 indels	89.3	3	2	No
AncDhaA18	Ancestor 0	30 mutations, 0 indels	89.5	3	4	Yes
AncDhaA19	Ancestor 18	49 mutations, 1 deletion	82.8	3	4	No
AncDhaA20	Ancestor 99	135 mutations, 6 indels	93.8	3	3	Yes

3. Manual curation of designs in round 3

In the initial two rounds of experiments, we selected a broad range of candidates from Model 1, focusing solely on their evolutionary trajectory profiles. For the candidates from Model 2 in the third round, we decided to additionally manually curate selected variants, pinpointing a few ancestors with notable characteristics. For instance, ancestor 3 stood out as its sequence was most similar to the wild type (WT), with a similarity of 97.93%, and it has the highest probability score from the VAE model at 99.24%. Ancestor 16 introduced the proline insertion at position 75 for the first time in the trajectory and showed a probability score of 95.6% and a WT similarity of 95.53% with 12 mutations. Ancestor 23 was the last ancestor whose sequence started with the regular pattern MSEIGT, closely matching the PDB sequence of 4WCV_A with an 88.31% similarity. This resemblance hints at the potential for good solubility and expression of the proposed sequence. Ancestor 23 had a model-assigned probability of 89.91%, featuring ins75P compared to the query and a total of 34 mutations.

We employed AlphaFold2 (as implemented by the Google Colab notebook [8]) to assess the anticipated structural impact of ins75P. The predicted structure was aligned with the original structure 4HZG (**Fig. S2**). The structural prediction revealed a shortening of the beta sheet caused by the L238F substitution. We therefore marked this mutation as potentially risky. We hypothesized that ins75P in ancestor 16 and the following ones was due to an artifact of the input MSA. In addition to the AlphaFold2 structure prediction, we analyzed scores returned by MutCompute [9]. The substitution P34V was assigned a low logarithmic ratio of -19.29 and was also marked risky. A summary of the MutCompute scores for substitutions in Ancestors 0, 3, 15, 16 is given in **Table S3**

In response to our findings, we assigned our designs to "safe" and "risky" categories. Ancestor 0 (AncDhaA13) was chosen as a negative control to explore the suspected adverse effects of the P34V and L238F mutations, incorporating all 8 mutations as suggested by the VAE model. AncDhaA14, deemed as the safest variant derived from ancestor 3, included only four mutations, none of which were considered risky. This variant allowed investigating the impacts of P34V or L238F mutations on protein folding individually. Another safe option was AncDhaA16 derived from ancestor 15, which included 9 mutations and excluded the risky P34V and L238F mutations. Finally, AncDhaA15 represented non-modified ancestor 16 design by the VAE, notable for including the proline insertion at position 75 alongside all 12 mutations, risk mutations included. AncDhaA17 was distinguished by ancestor 23 by removing risk mutations (P34T, L238F) and the proline insertion. AncDhaA17 had a total of 32 mutations.

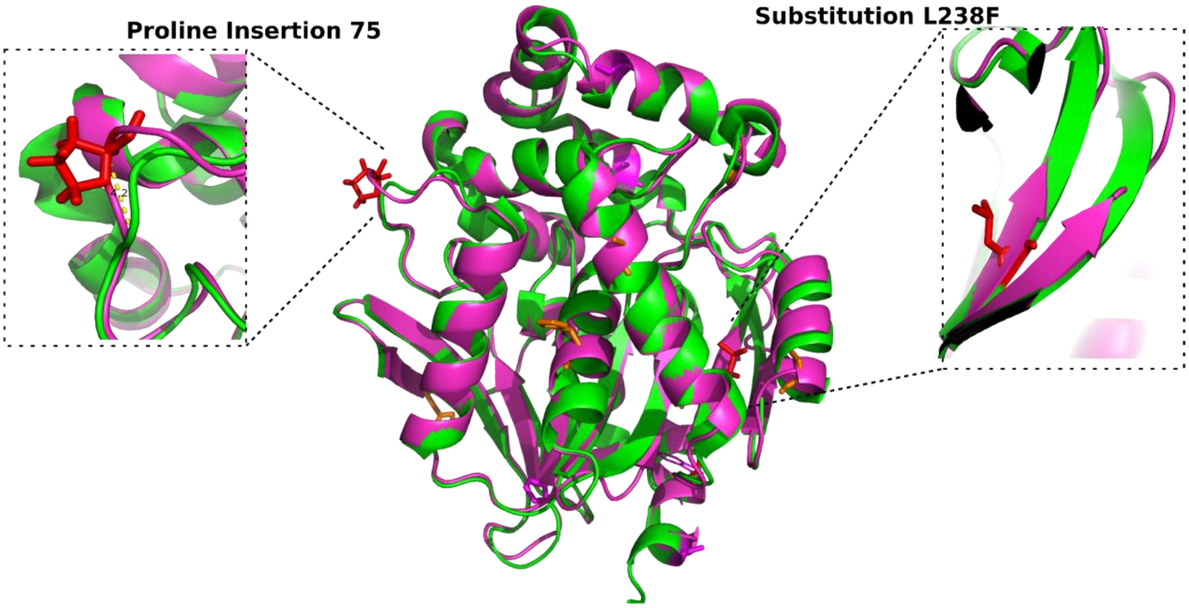

Fig. S2: AlphaFold predictions of AncDhaA15 (magenta) aligned to wild-type DhaA P0A3G3 (green). The structural impact of the proline insertion (on the left) and the L238F substitution (on the right) illustrate distinct changes to the protein's topology. The insertion of a proline results in the elongation of a loop, whereas the L238F substitution leads to a reduction in the length of the beta sheet.

Table S3: MutCompute scores of all mutations observed in AncDhaA13, AncDhaA14, AncDhaA15, and AncDhaA16. A negative score implies that the wild-type amino acid is predicted as more likely than the substitution.

Substitution	Score	Substitution	Score
H13K	-4.82	T155D	-5.21
P34V	-19.89	A172V	-3.25
M69F	-14.08	A220E	1.68
H114D	-5.66	L238F	-11.21
K124A	-14.39	S258W	-15.47
W141F	2.67	D280H	-9.4
P142V	-8.18	W289F	-8.39
A151K	-13.8	A292E	-1.94

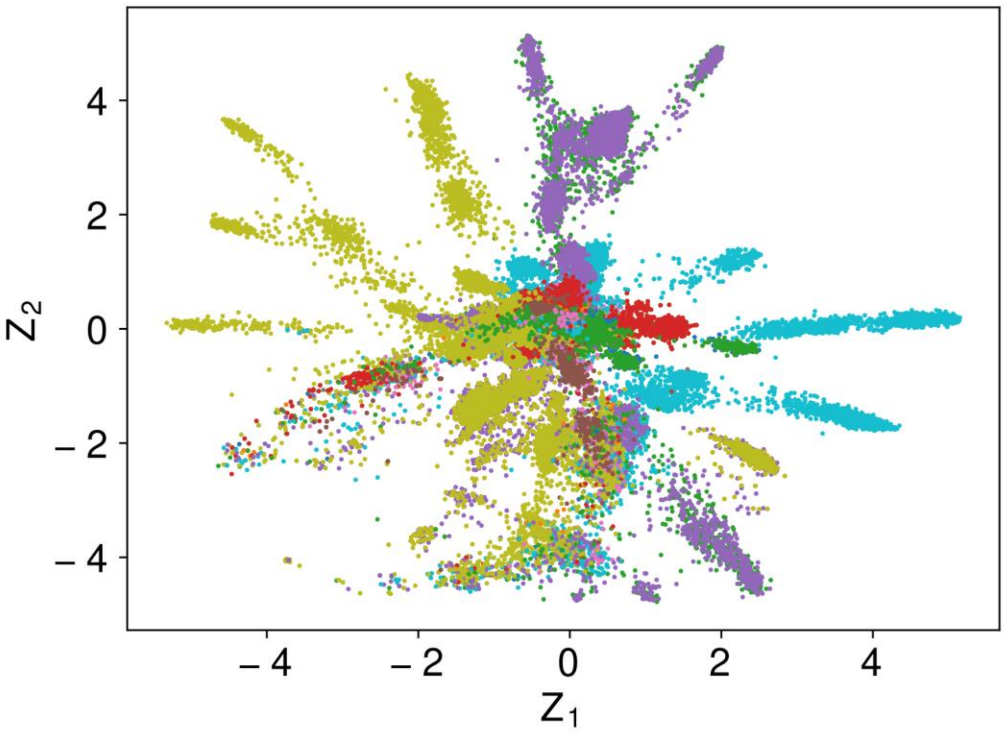

Fig. S3: Mapping of HLD I-IV phylogenetic tree branches into the latent space. We mapped the HLDI-IV dataset into the latent space, akin to the methodology described by Ding et al. [2]. We segmented the dataset into eight clusters, each aligning with the top eight branches of the phylogenetic tree constructed for the entire HLDI-IV dataset using FastTree software [10]. These clusters were then color-coded based on their branch membership. This mapping demonstrates the preservation of the spike-star distribution within the HLD I-IV dataset, indicating that the latent space encapsulates phylogenetic relationships.

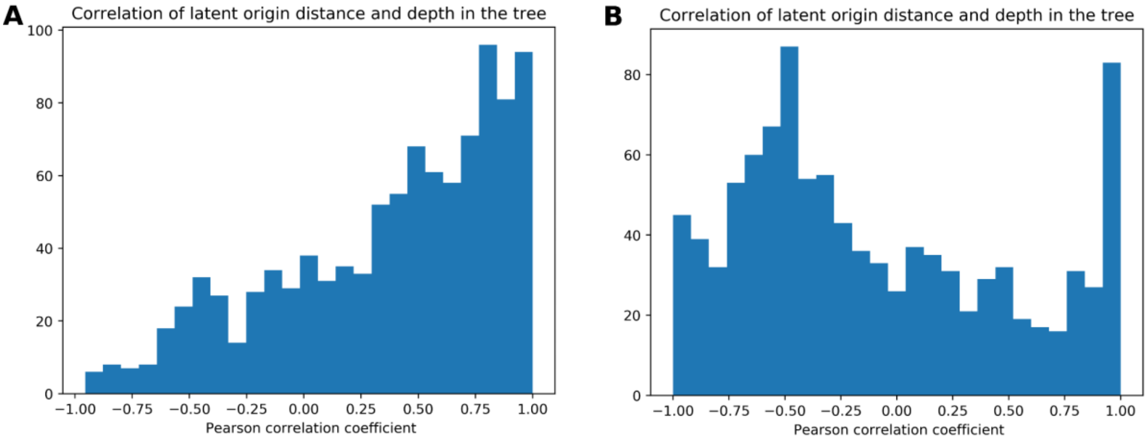

Fig. S4: Correlations between phylogenetic distances and the latent space coordinates. The histograms show the correlation values between the distance of the encoded sequence position from the center of the latent space and the depth of the corresponding node in the phylogenetic tree, for models with one deep layer (**A**) and two (**B**). The model with one dense layer can capture phylogenetic dependencies within the latent space structure compared to the model with deeper architectures.

4. Conditional variational autoencoders

In the conditional variational autoencoders (CVAE) [11], the VAE mechanism was extended with a tag added to the input of the encoder and decoder. Three tag values corresponding to the LOW, MEDIUM, and HIGH bins of sequence solubility values were used. The learning objective for CVAE can be interpreted as creating different representations of the latent space for each tag but with shared network parameters. Generating a new sequence from the latent embedding space can then be done for a desired tag (e.g. HIGH to maximize solubility), and the CVAE is forced to introduce the patterns observed for the corresponding solubility bin into the generated design.

The output of EnzymeMiner includes solubility labels predicted by SoluProt [7] for all output sequences in MSA. These values range from 0 to 1, with larger values corresponding to a higher probability of soluble expression in *E. coli*. The queried DhaA sequence (DhaA_S19) had a predicted solubility of 0.87. The solubility distribution of the HLD I-II dataset can be seen in **Fig. S6**. Most of the sequences had predicted solubility of less than 0.55. Therefore, the SoluProt tags were divided into three bins with thresholds set to make the distribution of sequences in each bin as uniform as possible (LOW: < 0.35, MEDIUM: 0.35-0.55, HIGH: > 0.55). A nearly uniform distribution for the solubility bins was chosen to encourage the CVAE to extract sequential patterns based on a balanced number of samples [12]. The sequences with corresponding tags are projected onto the latent space in the upper part of **Fig. S5.**

The latent space embeddings of CVAE do not provide tag separability in the latent space; instead, they introduce patterns to the reconstructed sequence corresponding to the average bin solubility values, as illustrated in **Fig. S5**.

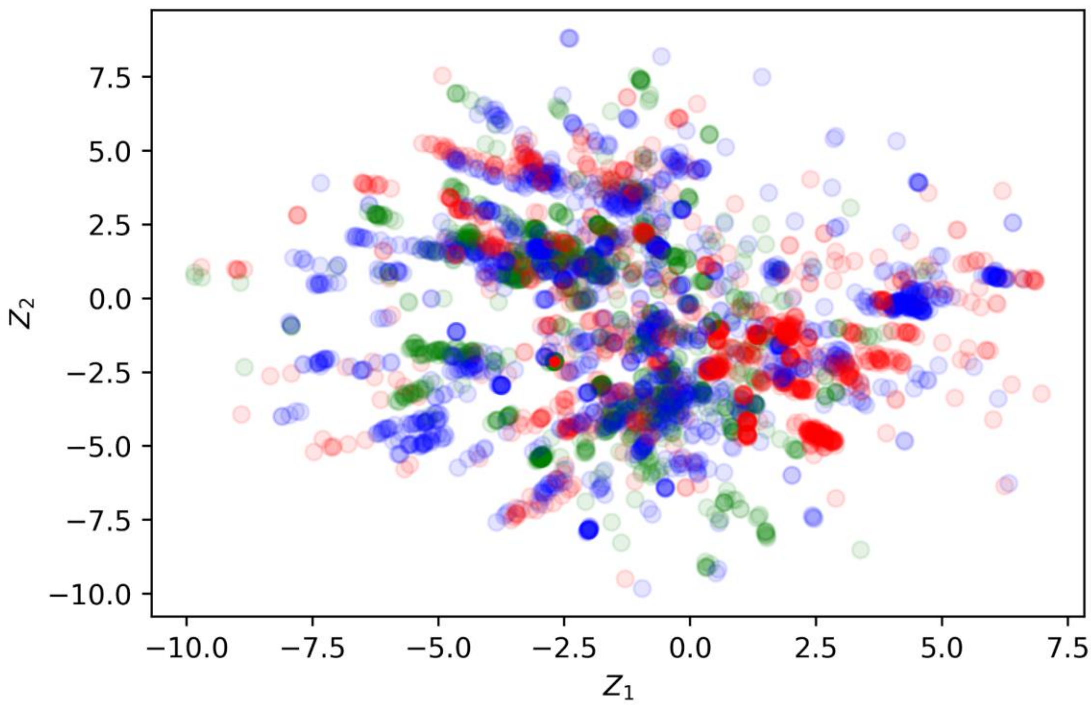

Protein	Solubility
DhaA_S19	0.659
ancestor_1	0.659
ancestor_20	0.624
ancestor_50	0.645
ancestor_80	0.333
ancestor_100	0.566
ancestor_18	0.602

Protein	Solubility
DhaA_S19_GREEN	0.810
ancestor_1	0.810
ancestor_20	0.761
ancestor_50	0.271
ancestor_80	0.443
ancestor_100	0.522
ancestor_99	0.560

Protein	Solubility
DhaA_S19_yel	0.786
ancestor_1	0.793
ancestor_20	0.598
ancestor_50	0.514
ancestor_80	0.208
ancestor_100	0.206
anc_23_yel	0.633

Fig. S5: The SoluProt solubility tag distribution in the latent space of conditional variational autoencoders and the effect of conditional labels on protein design. Top: Tag color corresponds to LOW (red), MEDIUM (blue), and HIGH (green). The latent space embeddings of CVAE do not provide tag separability in the latent space. Instead, they add patterns to the reconstructed sequence corresponding to the average bin solubility values. **Bottom-left:** CVAE model generates sequences with close to average HIGH bin solubility values in most cases. **Bottom-middle:** the VAE green model shows high solubility because it has learned to generate designs similar to PDB sequences. Despite the high solubilities for the first ancestors, the scores do not seem to be as stable as in the CVAE case. **Right:** the solubility values of the yellow model decrease as we move away from the query. **In the last row of each model**, the furthest straight-line designs selected for gene order are highlighted.

A

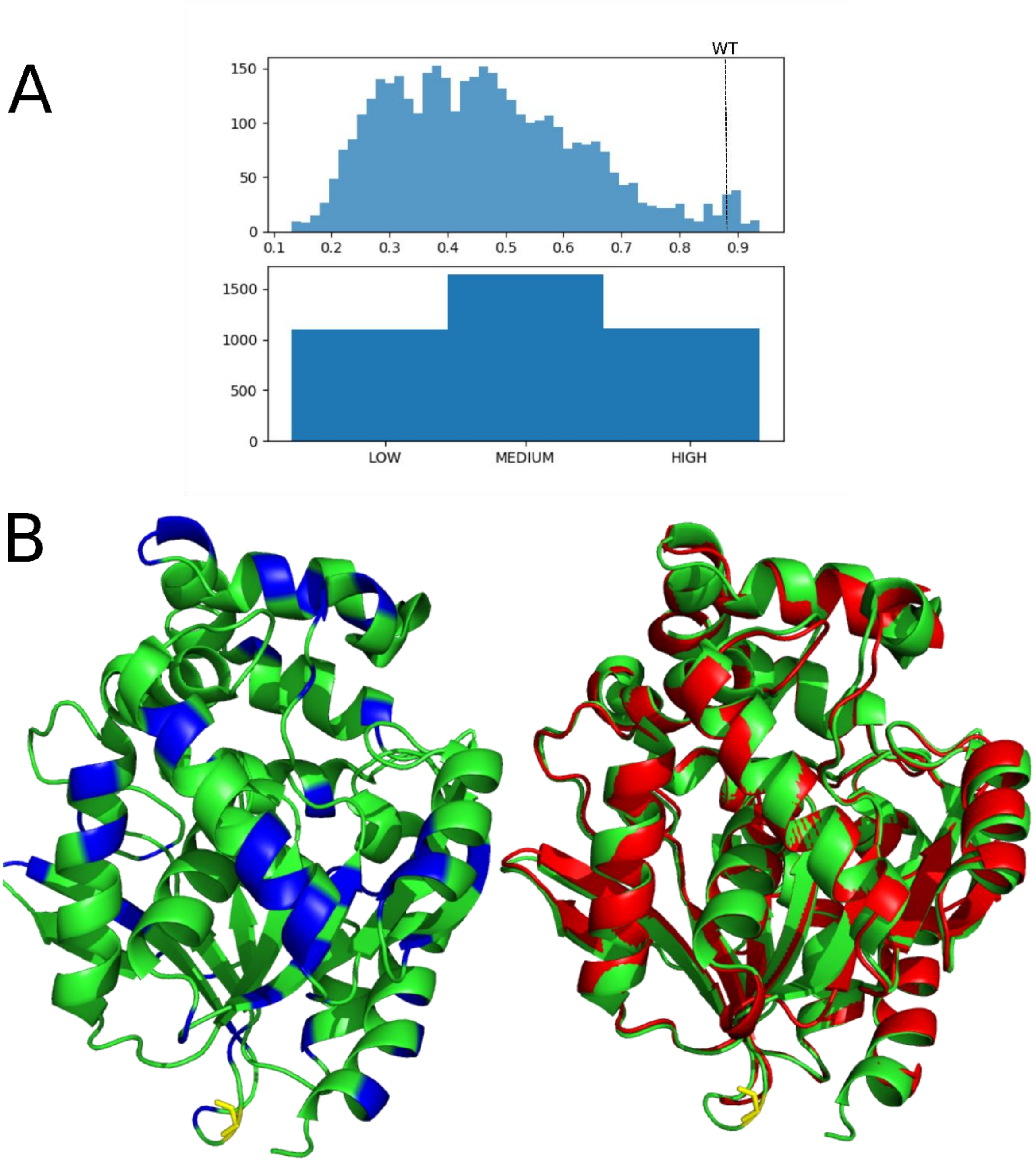

B

Fig. S6: The solubility distribution and proposed designs for the conditional variational autoencoder (Model 4). A Solubility distribution of the HLD I-II dataset predicted by SoluProt. The query sequence had a solubility value of 0.87 (dashed line). The scores were binned into LOW, MEDIUM, and HIGH using the cutoffs of 0.35 and 0.55 to produce the near-uniform distribution. **B Left:** DhaA wild-type structure 4hzg (green) with the highlighted mutations (blue) and deletion (yellow) introduced in AncDhaA19. **B Right:** AlphaFold2 prediction for AncDhaA19 (red) aligned to the original structure PDB ID 4hzg (green, RMSD = 0.499Å).

5. Model ensemble trajectories

Model ensembles are important in machine learning because they improve performance, enhance generalization capabilities, provide a more comprehensive representation of the data, and enable model selection and averaging. In our study, we examined the embeddings over an ensemble of four randomly initialized VAE models. Each individual model was used to generate 100 ancestral trajectories using a straight evolutionary strategy. These trajectories were then re-embedded into the latent space using the encoder weights of the first model (**Fig. S7**, left top). It is evident that the trajectories in the latent space exhibit scattered patterns, with limited spatial coherence observed between a few starting points from each trajectory. While the trajectory of ensemble model 0 is stable along the line to the origin, the other models tend to explore different sequential subspaces. These observations are consistent with the cross-embedding of evolutionary trajectories into different VAE models (**Fig. S7**).

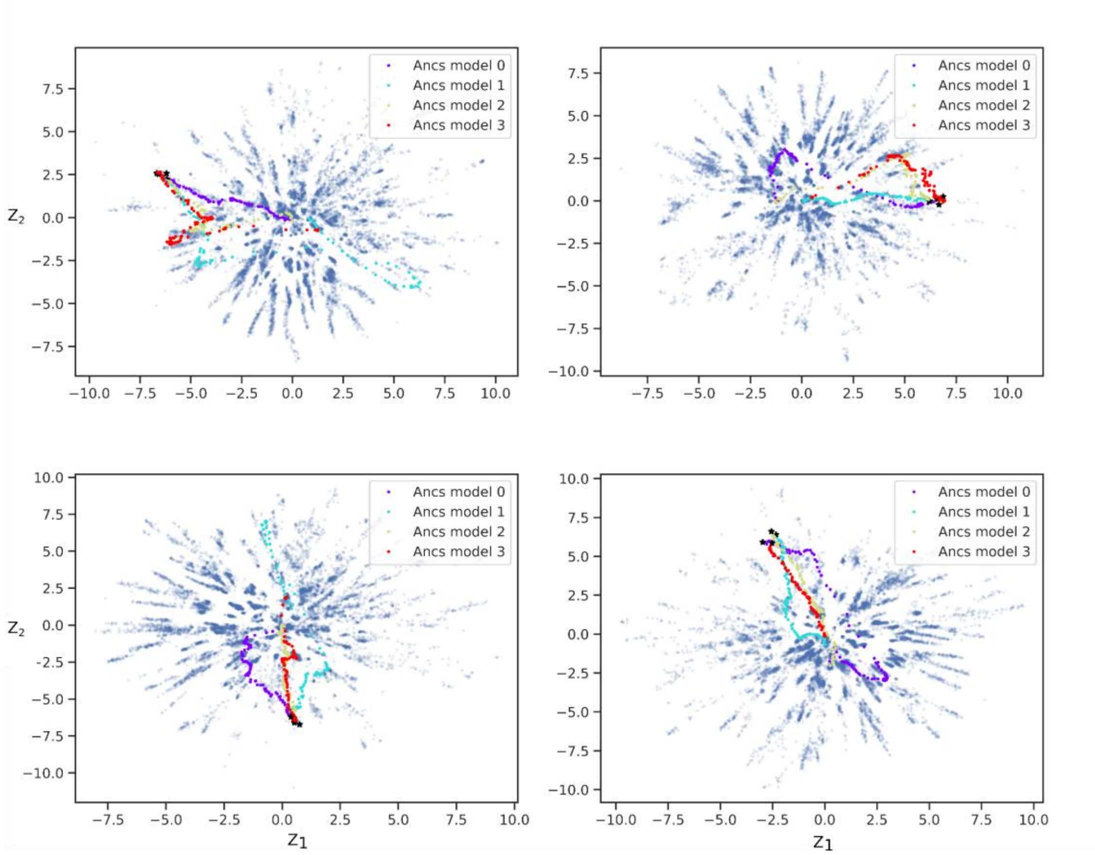

Fig. S7: Projection of trajectories onto different latent spaces (ensemble model 0: left top; ensemble model 1: right top; ensemble model 2: bottom left; ensemble model 3: bottom right)**.** Four variational autoencoder (VAE) models were randomly initialized and trained on distinct subsets of the same HLDI-IV dataset. The dataset itself was divided into five parts, with one part being excluded and the remaining parts used for training. Throughout all scenarios, the query sequence P59336_S14 was included in the training session. Each model generated 100 ancestral sequences using a straight evolutionary strategy, and these sequences were then embedded into the latent space of other models. Notably, we observed stability in the sequence embeddings only within the portion of the trajectory near the query embedding. However, discrepancies in the embeddings were observed in the central region, which may correspond to the high entropy of the decoder, as previously noted in [13]. These discrepancies could potentially serve as a distinct statistical metric in the sequence profile in future studies.

6. Investigation of mutational patterns in VAEs designs

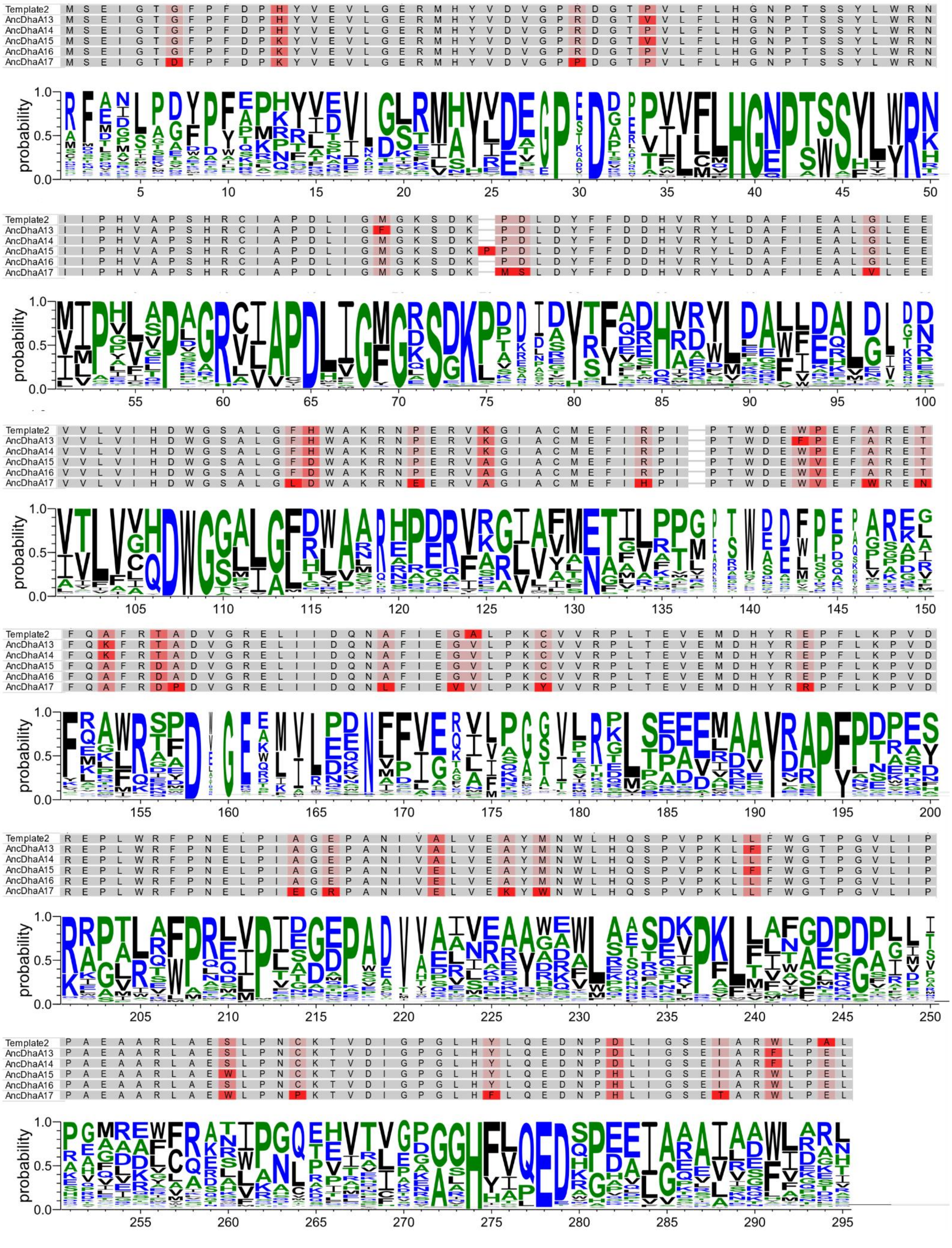

Fig. S8: **Highlighting Mutational Patterns on Sequence Logo of HLDI-II Model 2 Variants.** The MSA, visualized in NCBI MSA Viewer, displays the evolutionary relationships between template sequence and designed variants (AncDhaA13-17) generated using VAEs Model 2. The sequence logo, created by WebLogo 3 tool ([22], https://weblogo.threeplusone.com/) below the MSA visualizes the conservation and variability of amino acids across aligned positions, with the height of each letter representing the relative frequency of that residue at a given position. Positions with mutations in the designs are highlighted in red in the MSA. Mutations predominantly

occur in positions with moderate to low conservation, indicating the model's preference for targeting more variable regions. Certain mutations introduced in earlier designs are preserved in subsequent designs with higher sequence numbers, indicating their retention throughout evolution. In contrast, some mutations are reverted in later designs, reflecting an iterative refinement process where the model explores different solutions. These reversion events suggest that competing selective pressures or structural constraints may favor the original residue in certain positions. Please note two gaps in the query sequence introduced by the MSA preprocessing step. These gaps result in a discrepancy between the main text (e.g., the risky mutation L238F in the query sequence) and the sequence profile, which shows this mutation shifted to position 240.

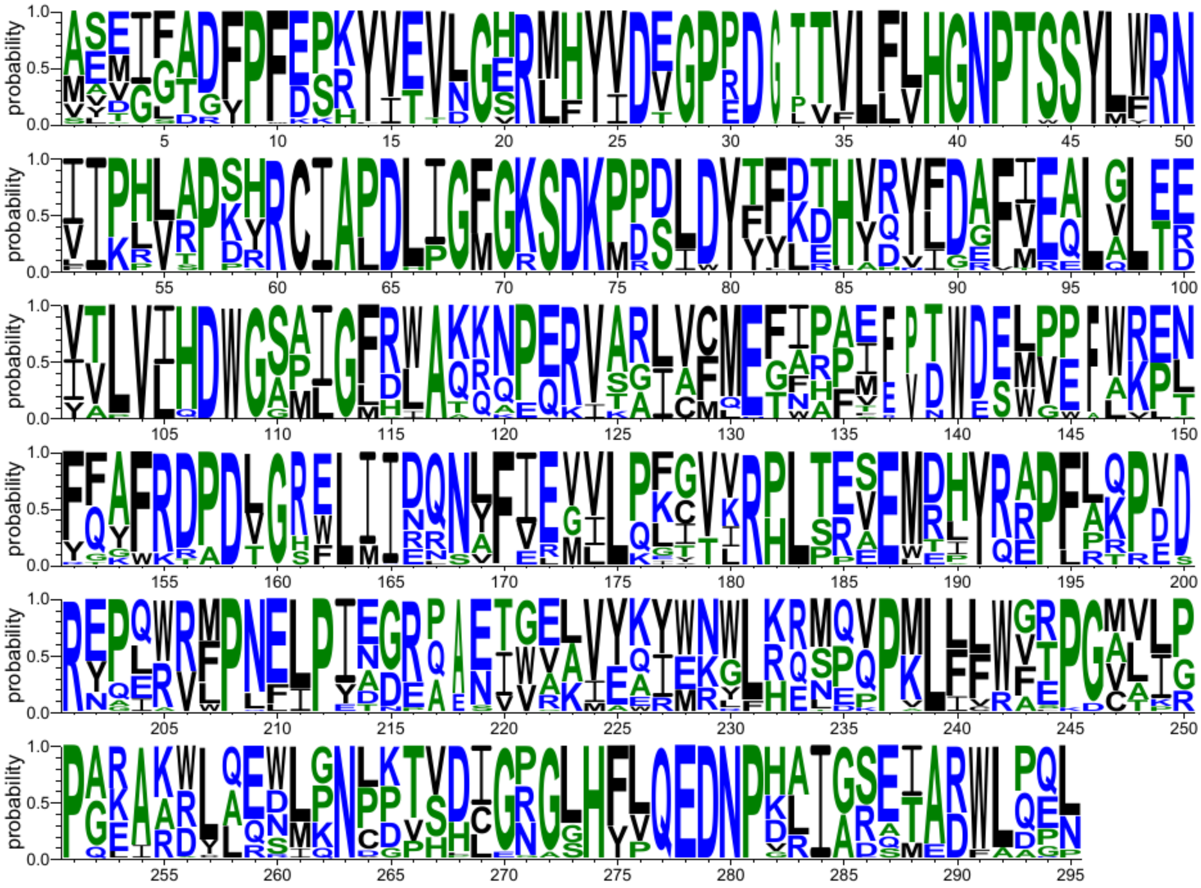

Fig. S9: Profile of 100 generated Model 2 ancestors. The ancestors generated from VAE Model 2 were aligned to analyze mutational patterns. An examination of the mutations across all 100 designs reveals the accumulation of individual mutations at specific positions along an evolutionary trajectory. The overall profile of the generated ancestors differs from the multiple sequence alignment (MSA) profile (**Fig S14**), indicating that the design process of the VAE diverges from simply converging on a consensus sequence. This reflects a change in the selection criteria.

7. Variant production

In the first round, the solubility of DhaA ancestor variants was analyzed after their overproduction in *E. coli* and purification, where only AncDhaA1 was purified as a soluble protein in sufficient yield (**Fig. S10**). This variant was also the only active one within the screening of the dehalogenase activity in whole cells using a halide oxidation (HOX) assay (**Fig. S11A**) [14]. The variants produced in the first round showed overall low solubility, consistent with outcomes from several previous machine learning-based pipelines for protein design, achieving a solubility success rate of around 20% [15,16].

In the second round, the ancestors 5, 7, and 8 (AncDhaA10-12) were selected for production to stay closer to the template. Among these variants, AncDhaA10 showed the highest expression, followed by AncDhaA11, while AncDhaA12 exhibited the lowest expression and solubility (**Fig. S10B**). AncDhaA10 was also shown to be active by HOX assay (**Fig. S11B**). Based on the results from both rounds, AncDhaA1, AncDhaA10, and AncDhaA11 were chosen for further biochemical characterization.

From the third round of designs, six variants showed average or high solubility and only AncDhaA17 and AncDhaA19 were poorly soluble in the tested buffer. The HOX assay revealed two highly active variants AncDhaA13 and AncDhaA14 with activities comparable to templates and positive controls (**Fig. S11B**). Variants AncDhaA16 and AncDhaA20 exhibited lower activity, comparable to that of AncHLD-RLuc [17] (**Fig. S11B**), while AncDhaA15 and AncDhaA18 showed low activities (**Fig. S11B**).

7.1. Small-scale protein over-expression and affinity purification test

Several *E. coli* colonies were streaked to inoculate 2 ml of starting media (2xLB supplemented with 0.5% glucose and 100 μg/ml ampicillin) in a 24-deep-well plate (GE Healthcare, UK). The plate was covered with an air-pore membrane and incubated at 37°C for 4 hours, 200 rpm. After incubation, 2 ml of induction media (2xLB supplemented with 0.6% lactose, 50 mM HEPES (pH 7.4), 0.5 mM IPTG, and 100 μg/ml ampicillin) was added. The plate was covered with an air-pore membrane and incubated at 22°C for 16 hours, 200 rpm. Cells were harvested by centrifugation using Sigma 6K-15 centrifuge (SciQuip, UK) for 10 min, 1519 g, and 4°C, and resuspended in 1.3 ml of a purification buffer (16.4 mM K_2HPO_4, 3.6 mM KH_2PO_4, 400 mM NaCl, 10 mM imidazole, pH 7.5). After cell disruption (Sonic Dismembrator Model Q700S, FisherBrand, USA) the whole soluble fraction was clarified by centrifugation for 20 min, 3572 g, and 4°C. Soluble fraction was added to TALON SuperFlow Metal Affinity Resin (Takara) pre-equilibrated with sterile water and incubated for 2 hours on a roller (40 rounds/min) at 4°C. Unbound proteins were washed twice by and centrifuged at 94 x g for 2 min followed by resuspending in a purification buffer. After the second wash, 40 μl of SDS-PAGE loading buffer (2x Laemmli Sample buffer containing DTT) was added to each protein/resin sample. Samples and a marker (Color Prestained Protein Standard, Broad Range 10–250 kDa, New England Biolabs, USA) were loaded on SDS-PAGE gel with run conditions: 400 mA, 200 V, 40 min. After staining with InstantBlue™ (Missouri, USA) for 20 min, the gel was washed with water for 40 min.

7.2. Cell cultivations for enzymatic screenings and HOX assay

Single colonies of transformed cells were transferred into sterile 96-well plates (MTP) containing 100 μl of LB medium supplemented with ampicillin (100 μg/ml). The plates were covered with air-pore membrane and cultivated for 3 h at 37°C and 200 rpm. After that, an additional 100 μl of LB medium with ampicillin (100 μg/ml) and IPTG (1 mM) were added to the mini-cultures and MTP was afterward incubated at 20°C, 200 rpm, for 18 hours. Cell cultures were harvested by centrifugation at 4°C, 1600 x g, 20 min. The supernatant was discarded, and the cell pellets were washed with 200 μl of

reaction buffer (1 mM orthovanadate, 20 mM phosphate buffer, pH = 8.0). MTP was centrifuged again at 4°C, 1600 g, 20 min and the washing step was repeated twice. Finally, the pellets were resuspended in 200 µl of the reaction buffer, and optical density (OD_{600}) was determined spectrophotometrically. Into each well of a new black bottom 96-well MTP plate, 100 µl of MasterMix was dispensed: 25 µM aminophenyl fluorescein, 26 mM H_2O_2, 1.1 U *Curvularia inaequalis* vanadium chloroperoxidase with additional His tag, 1 mM orthovanadate, 20 mM phosphate buffer, pH = 8.0. Also, 4 µl of resuspended cells were added into each well, followed by the addition of 96 µl of 0.3 mM 1,2-dibromoethane in the reaction buffer. Fluorescence was measured using Synergy™ H4 Hybrid Microplate Reader (BioTek, USA) (Excitation at 488 nm; emission detection at 525 nm; 30°C). Data for all tested variants were measured in four biological replicates and average activity with a standard deviation of four measurements was determined.

7.3. Large-scale protein over-expression

Several colonies of *E. coli* were incubated in 10 ml 1x LB medium supplemented with 100 µg/ml ampicillin. The pre-culture was incubated at 37°C for 4 hours. After incubation, the pre-culture was added to 1 liter of 1x LB medium supplemented with 100 µl/ml ampicillin. The culture was incubated to OD_{600} = 0,8 and expression was induced by the addition of IPTG to a final concentration of 0.5 mM. The cell culture was incubated at 20°C, 150 rpm, 16 h and harvested by centrifugation at 4000 rpm, 4°C, 25 min. The cell pellet was resuspended in approximately 30 ml of harvesting purification buffer A: 16.4 mM K_2HPO_4, 3.6 mM KH_2PO4, 400 mM NaCl, 10 mM imidazole, pH 7.5, and frozen at -70°C.

7.4. Protein purification with metal affinity resin

The DNase was added to the cell culture (20 µg/ml) after defrosting from -80°C. The culture was sonicated (Sonic Dismembrator Model 705 Fisher Scientific, USA) in 6 x 2-minute cycles with a 50 % amplitude (5 s pulse, 5 s pause). The cell suspension was centrifuged (21036 g, 4°C, 1 h) using a Sigma 6-16K centrifuge (SciQuip, UK) equipped with the 12166 rotor. 1 liter of cell-free extract was divided into two 50 ml conical centrifuge tubes with washed Resin (TALON® Superflow Metal Affinity Resin, Takara). The mixture was incubated for 1.5 hours at 4°C on a roller (40 rounds/min). After incubation, the resin with bound proteins was centrifuged in a pre-cooled centrifuge for 10 min, 130 g, 4°C using a Sigma 2-16K centrifuge (SciQuip, UK) equipped with the 11192 rotor. The supernatant was discarded, and harvesting buffer A (16.4 mM K_2HPO_4, 3.6 mM KH_2PO_4, 400 mM NaCl, 10 mM imidazole, pH 7.5) was added and transferred to a gravity-flow column equilibrated in the same buffer. The column with mixture resin protein was washed with approximately 200 ml of harvesting buffer A, and a 50 mM phosphate buffer (pH 7.5) was gradually added to the column. The protein was eluted from the resin using an elution buffer of 50 mM phosphate buffer, 300 mM imidazole (pH 7.5).

7.5. Purification by gel filtration on FPLC

Affinity-purified proteins were purified in the second step by gel filtration on ÄKTA Pure™ (Cytiva, USA) equipped with HiLoad 16/600 Superdex 75 pg column. After column equilibration with 50 mM phosphate buffer (pH 7.5), proteins were purified using the same buffer and concentrated on an Amicon® Ultra-15 Ultracel-10 gravity flow column 10K (Merck Milipore Ltd.).

7.6. Dehalogenase activity measurements on MicroPEX

The droplets were generated using Mitos Dropix (Dolomite, UK). A custom sequence of droplets (150 nl aqueous phase, 300 nl oil spacing) was generated using negative pressure (microfluidic pump).

The droplets were guided through a polythene tubing to the incubation chamber. Within the incubation chamber, the halogenated substrate was delivered to the droplets via a combination of microdialysis and partitioning between the oil (FC 40) and the aqueous phase. The reaction solution consisted of a weak buffer (1 mM HEPES, 20 mM Na_2SO_4, pH 8.2) and a complementary fluorescent indicator 8-hydroxypyrene-1,3,6-trisulfonic acid (50 μM HPTS). The fluorescence signal was obtained using an optical setup with an excitation laser (450 nm), a dichroic mirror with a cut-off at 490 nm filtering the excitation light, and a Si-detector. By employing a pH-based fluorescence assay, small changes in the pH were observed and enabling monitoring of the enzymatic activity. Reaction progress was analyzed as an end-point measurement recorded after the passing of 10 droplets/sample through the incubation chamber. The reaction time was 4 min. The raw signal of every single measurement was first processed by the in-house LabView-based (National Instruments, USA) software MicroPEX Data Analyzer 1.0. The peaks were assigned to the particular sample, and the mean signal was calculated for them. The output XLS file gathering mean signal values for every sample type (calibration, enzyme activity, buffer, blank 3 buffer, and blank enzyme) for the dataset served as an input for the MatLab (Mathworks, USA) script to calculate specific activities using the same principle as for previous measurements [6,18]. The activities were classified as "not determined" whenever the measured product concentration was below the limit of detection (LOD – 3 times the standard deviation of the noise signal). Each substrate had a different calibration curve, so the LOD product concentration was in the range of 10-100 μM).

The matrix containing the activity data of 32 previously identified HLDs [6] and the activities obtained for all variants in this study (all measured on MicroPEX) was analyzed by PCA in MATLAB (MathWorks, United States) to uncover the relationships among individual HLDs (objects) based on their activities toward the set of halogenated substrates (variables). Two PCA models were constructed to visualize systematic trends in the dataset. The first one was done on the raw data, which ordered the enzymes according to their total activity. The second PCA was carried out on the log-transformed data after adding 1 to each specific activity to avoid taking the logarithm of zero. The resulting values were then divided by the sum of the values for a particular enzyme. These transformed data were used to calculate principal components, and the components explaining the highest variability in the data were then plotted to identify substrate specificity groups.

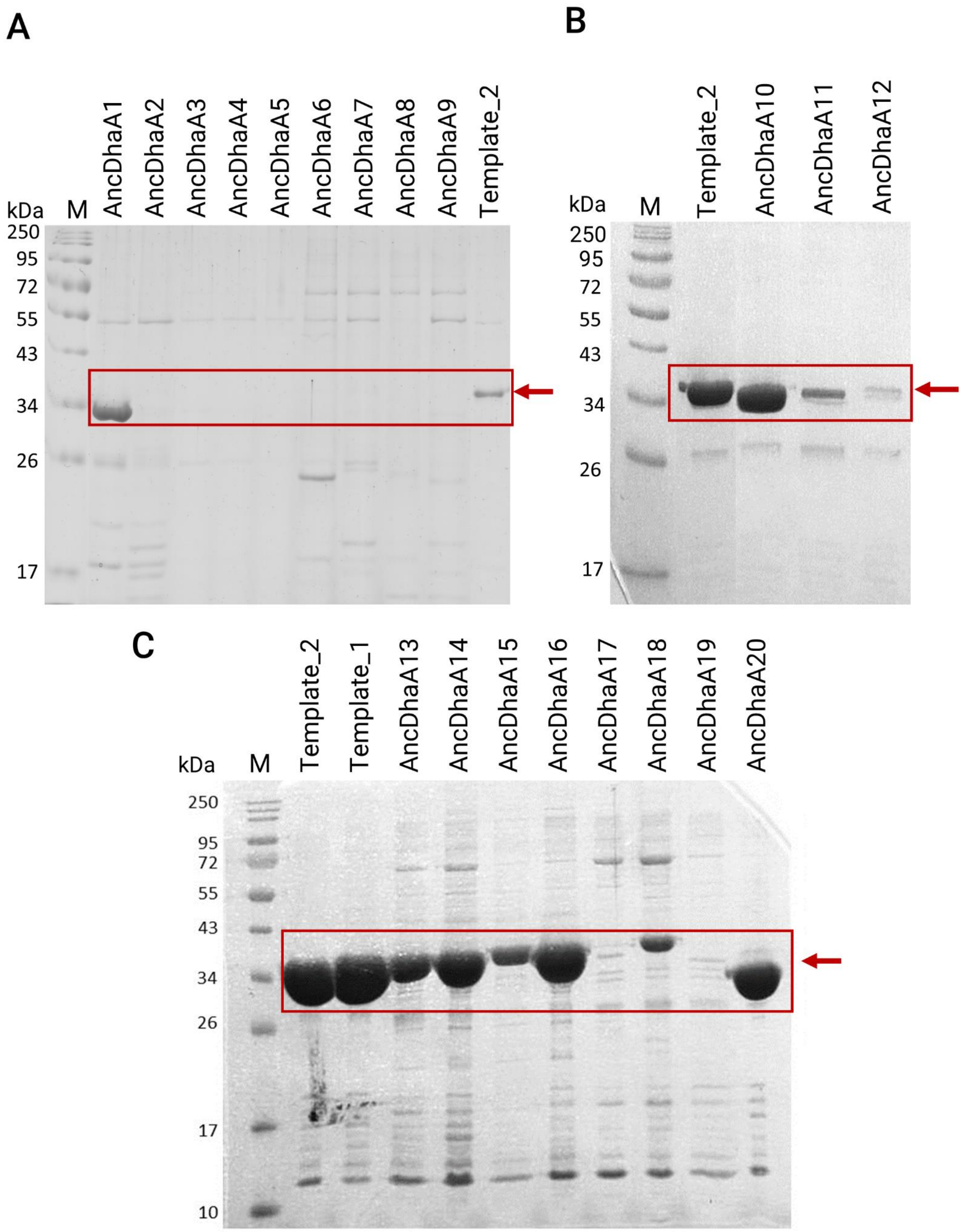

Fig. S10: Solubility screening of tested variants using SDS-PAGE. SDS-PAGE gels of affinity-purified ancestor variants from first (**A**), second (**B**) and third rounds (**C**). M indicates markers.

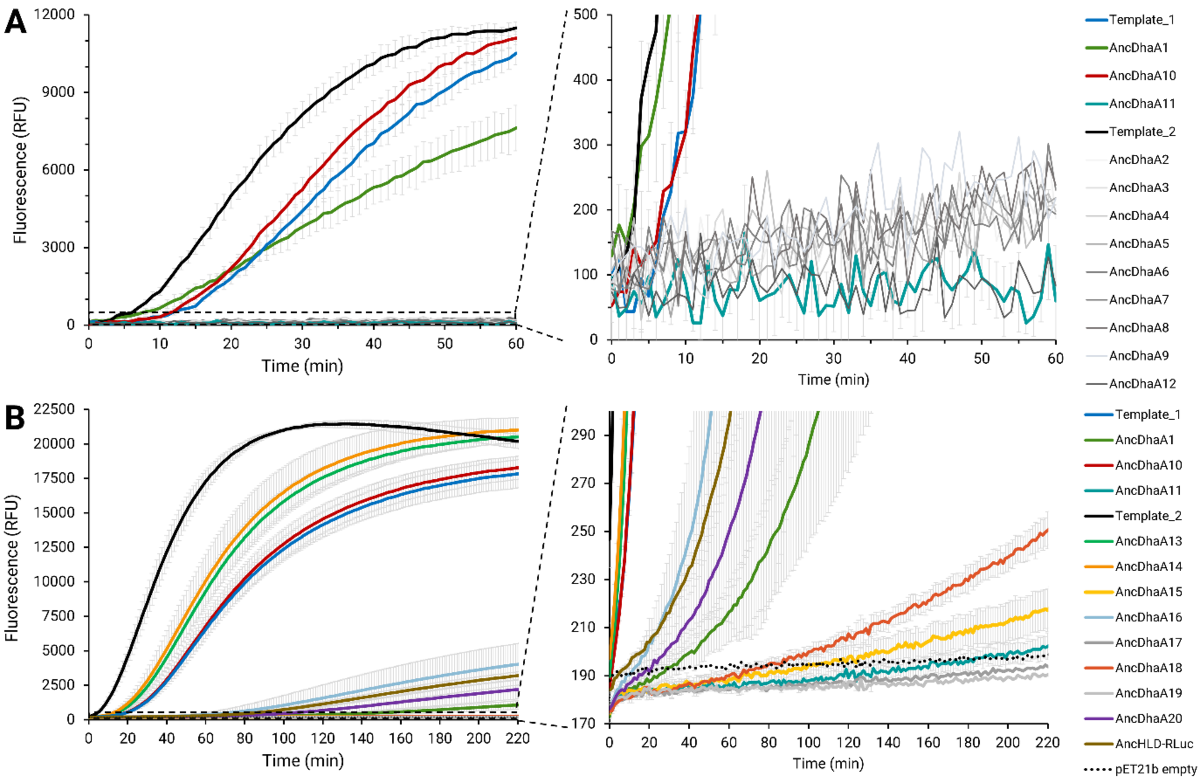

Fig. S11: Screening of dehalogenase activity by HOX assay. Whole-cell activity screening with 1,2-dibromoethane. **A** Variants from the first two rounds. **B** Variants from the third round augmented by soluble variants from the first two rounds. Dashed frames (left) represent the graphs on the right. Insoluble variants are shown in shades of grey.

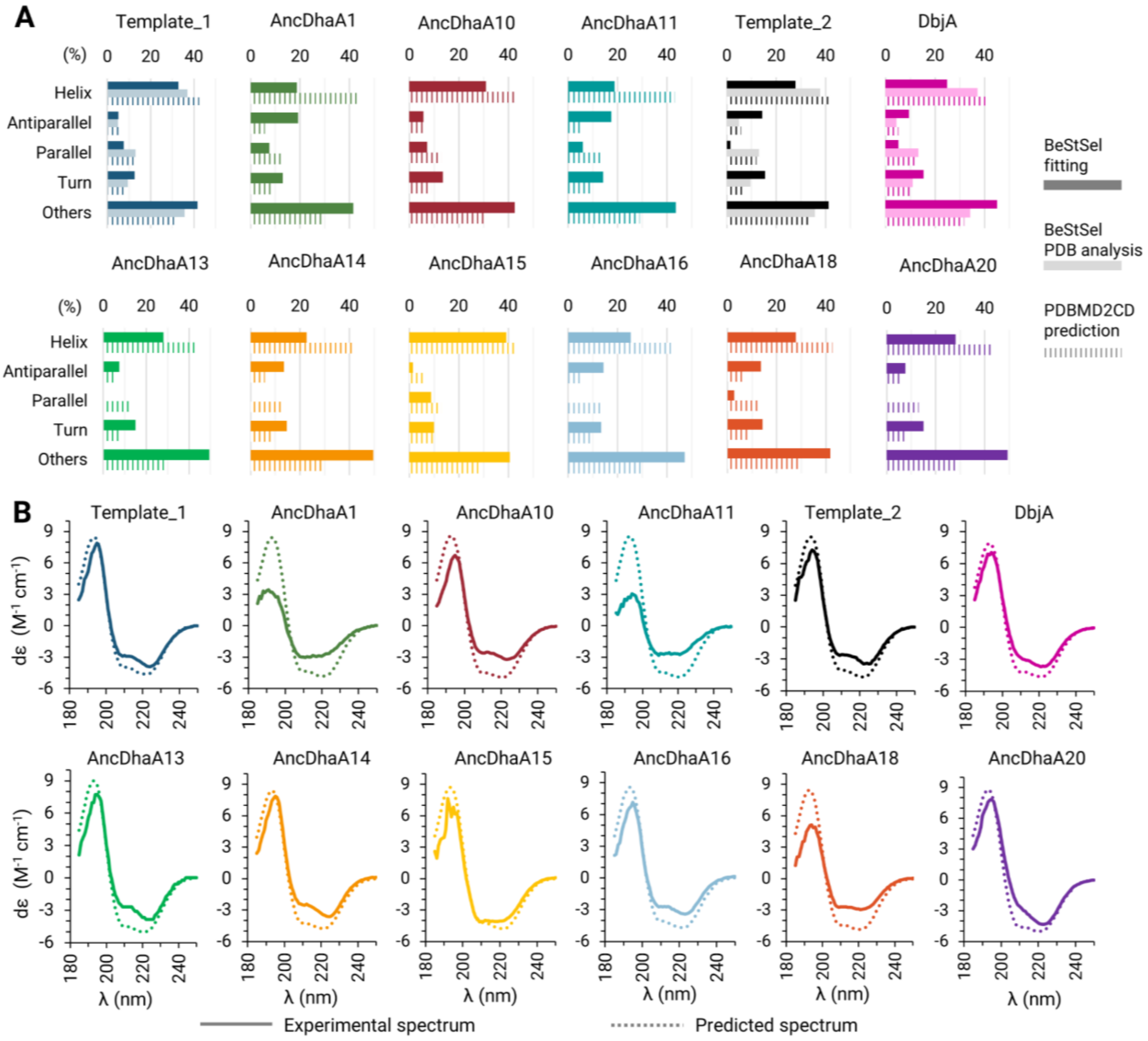

Fig. S12: Secondary structure analysis. A The secondary structure composition (in %) of individual variants evaluated by fitting of experimental data (solid fill, full color) or by analysis of PDB structures (solid fill, lighter color, only for Template) by BeStSel [19]tool, and predicted by the PDBMD2CD [20] tool based on PDB structures or AlphaFold models (diagonal stripes). **B** The comparison of predicted (dotted line) and experimentally determined (solid line) CD spectra for individual variants.

Table S4: Thermostability measurements. The thermostability parameters were determined for all variants using nanoDSF, except for AncDhaA11, marked by *, where relevant data could be collected only by circular dichroism.

**Variant**	**Sequence identity[1] (%)**	T_{on} **(°C)**	T_m^{app} **(°C)**	T_{agg} **(°C)**
Template 1	–	43.8 ± 0.0	53.0 ± 0.0	–
AncDhaA1	84.9	39.3 ± 0.3	49.1 ± 0.1	45.5 ± 1.4
AncDhaA10	97.6	41.2 ± 0.2	50.4 ± 0.0	–
AncDhaA11*	94	–	53.9 ± 0.6	–
Template 2	–	40.1 ± 0.1	50.6 ± 0.3	–
AncDhaA13	97.2	47.1 ± 0.0	57.7 ± 0.0	–
AncDhaA14	98.5	43.6 ± 0.1	56.1 ± 0.0	–
AncDhaA15	95.5	35.2 ± 0.7	47.8 ± 0.2	–
AncDhaA16	96.8	26.0 ± 4.3	53.9 ± 0.6	–
AncDhaA18	89.7	35.8 ± 0.1	45.4 ± 0.2	–
AncDhaA20	51.4[2]	52.1 ± 0.1	59.6 ± 0.0	48.5 ± 0.3

[1] Sequence identity to the respective template.

[2] AncDhaA20 shows 93.8% sequence identity to other haloalkane dehalogenase, DbjA.

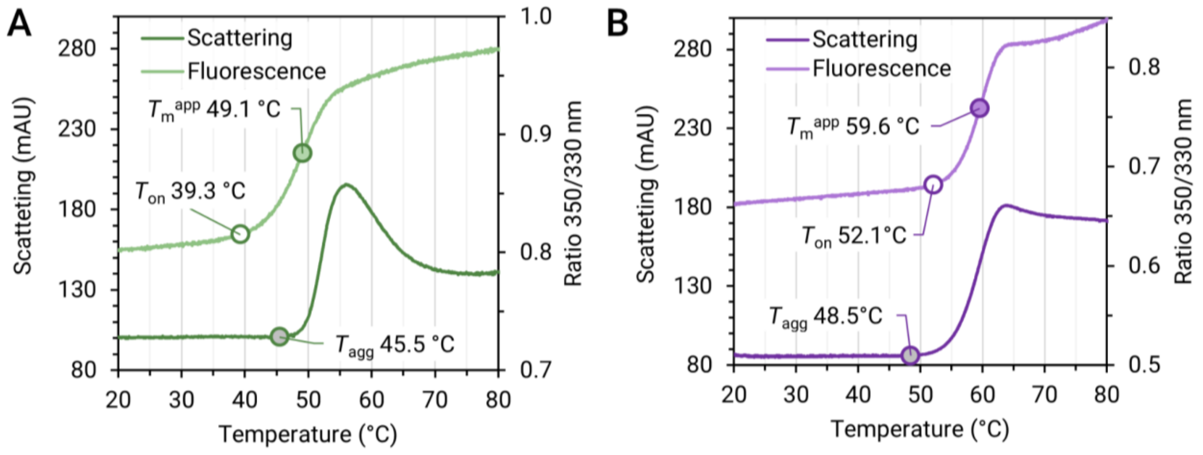

Fig. S13: Aggregating variants studied by static light scattering. The graphs show the dependencies of scattering (darker color) and fluorescence (lighter color) for AncDhaA1 (**A**) and AncDhaA20 (**B**). Aggregation onset, denaturation onset, and apparent melting temperatures are highlighted.

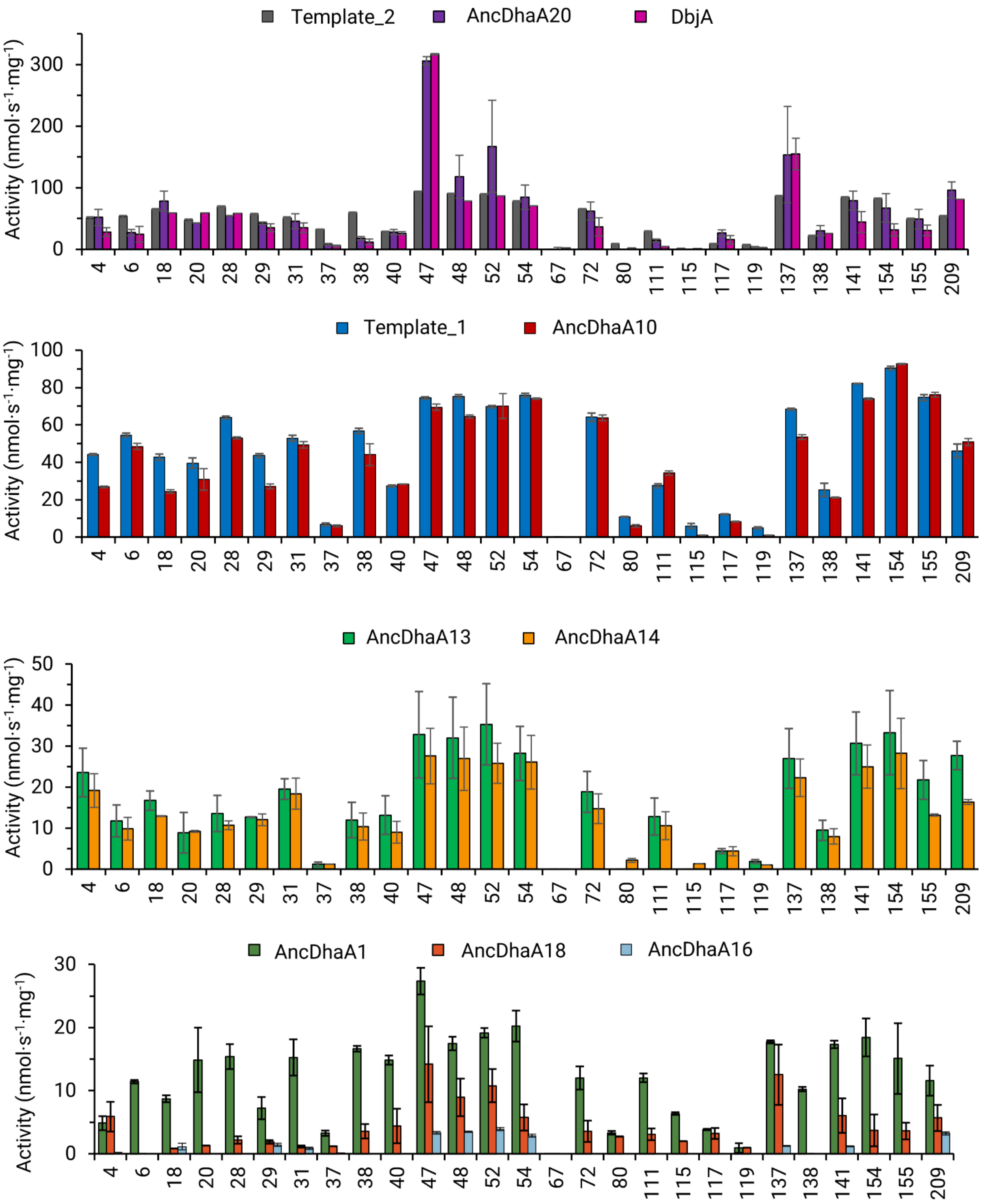

Fig. S14: Substrate specificity of selected designs determined by microfluidics. Substrate specificity profiles for the variants. Numbers on the X-axis denote codes for substrates established previously [21].

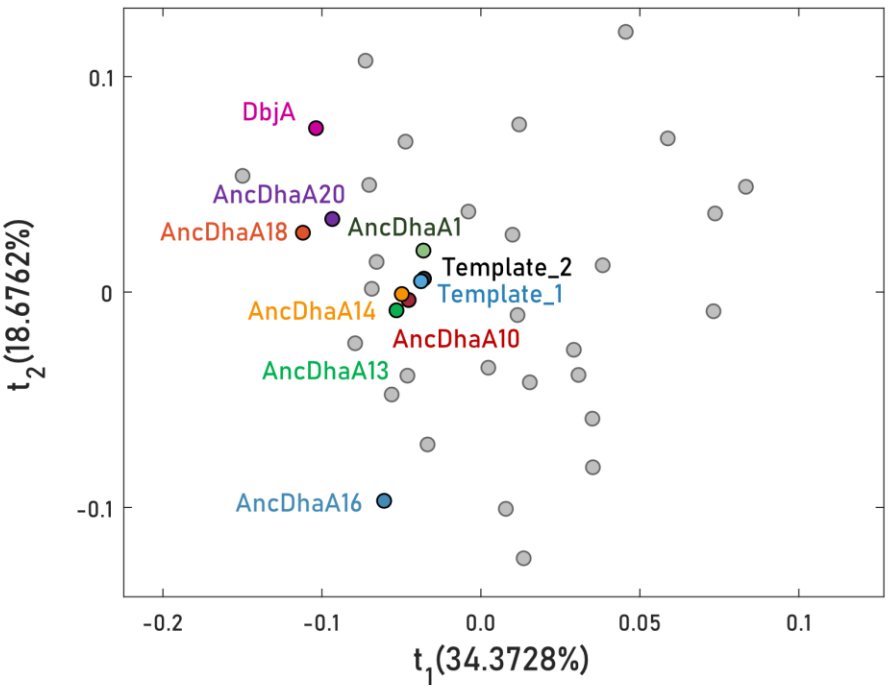

Fig. S15: **Principal component analysis of substrate specificities.** The displayed data include template proteins (Template_1 and Template_2), benchmark dehalogenase DbjA and designed variants (AncDhaA). The t_1/t_2 score plot (log-transformed data) describes 53% of the variance in the dataset. The benchmark dehalogenases, measured previously [6,18], are shown in gray. The newly characterized variants within this study are highlighted.

8. List of generated sequences

>**Template1** (P59336, 294 positions)

MSEIGTGFPFDPHYVEVLGERMHYVDVGPRDGTPVLFLHGNPTSSYLWRNIIPHVAPSHRCIA
PDLIGMGKSDKPDLDYFFDDHVRYLDAFIEALGLEEVVLVIHDWGSALGFHWAKRNPERVK
GIACMEFIRPIPTWDEWPEFARETFQAFRTADVGRELIIDQNAFIEGVLPKCVVRPLTEVEMDH
YREPFLKPVDREPLWRFPNEIPIAGEPANIVALVEAYMNWLHQSPVPKLLFWGTPGVLIPPAE
AARLAESLPNCKTVDIGPGLHYLQEDNPDLIGSEIARWLPGLA

>AncDhaA1

MSSIGAGCPFDPHYVEVLGSRMHYVDEGPREGAPVLFLHGNPTSSYLWRNIIPHVAPLHRCIA
PDLIGMGKSDKPDLAYRFDDHVRYLDAFIEALGLERVVLVIHDWGSALGFHWAHHHPERVK
GIVFMEFIRPFETWDDWPEFARETFQAFRTPGVGEELVLDQNAFIEGVLPKCVVRPLSDVEMD
HYRAPFRKPASRKPLLRFPREIPIAGQPANVTALVEGYLDWLTQSPVPKLLFWGHPGVLIPPEE
AARYAESLPNCKVVDIGPGLHYLQEDNPDLIGREIARWLPGLA

>AncDhaA2

MTSISVECPFEHHYVEVLGSRMHYVDEGEGAPVLFLHGNPTSSYLWRNVIPHVRGLGRCIAP
DLIGMGASDKPDLSYRFDDHARYLDGFIEALGLERVVLVIHDWGSALGFDWAHHHPERVKG
LVFMEAIRSPLASWQDFPERARGLFQGFRTPGVGEDLVLDQNAFIEQVLPKCVVRPLSPAEM
DHYRAPFPDPTSRKPLLRFPREIPIAGQPVDVTRIVEGYRDWLCHSPVPKLLFAGHPGVLIPPP
AVAWYREHLPNCEVVDIGRGLHFLQEDNPDLIGRGIAKWMGLA

>AncDhaA3

MTAISADFPFAVRDVQVHGSRMAYIDEGSGDPIVFLHGNPTSSYLWRNVIPHVRDVGRCIAP
DLIGMGASDKPDIDYRFVDHALYLDGFISALGLNRITLVVHDWGSALGFDWAMRHESRVRG
VAFMEAFLGPVSWEQFTEQGREIFQKFRTPGVGETMVLDHNMFVEQVLPGSVVRSLSPAEM
QHYRAPFVERTARKPMLAWPREIPIAGEPADVVTVVSRYRYALCQSQVPKLLFTVEPGVLVR
APLVAWCREHLAKLDVIALGRGLHFLQEDHPHEIGEGIADWVRRTP

>AncDhaA4

QQAISARFPFMQTIEVLGSRMHYVDTGGDVPVVFLHGNPTSSYLWRNVIPHVSDGGRALAPD
LIGMGASGKPDSDYRFVDHARYLDAFLDALGLRVTLVVHDWGSALGMDWAARHPDRVRGI
AFMEAFLRPVAWSQVPPQGAELFRKFRSPGEGETMVLEQNMFVEGLLPASIRSLSAAEMDAY
RAPYPTPESRKPMLAWPREIPIAGEPADTVTIIEKYAYWLCQSAVPKLLFHVEPGVLLRPEVA
DWAANTFAKLEARNIGPGAHFCQEDRPEAIGEAIADWVRRHA

>AncDhaA5

MPTVEVLDSFIYYRDTGGGIPVVFLHGNPTSSYLWRNVIPHVAGTRALAPDLIGMGASGKPDI
DYRFADHARYLDAWFDALGLRVILVGHDWGGALGMHYAARHPGRVRGIALIETFLRPMDW
DEMPPQGAELFRAFRSPEGEEMVLEQNLFIEFNLPKLVRSLSAADLDAYRAPFPTPESRKPVL
VWPREFPIDGEPADVVAIVREYGAWLAAPEVPKLLMHVEPGVGLPEVADWAATTFANLEAF
IGPAGHHCPEDQPEAIGRAIADWLRRHA

>AncDhaA6

MVLGTFLHYVEAGTSPVVFLHGNPTSWYVWRHVMPLLAYRCLAVDLRGFGQSGKPEAYRL
VDHAEDLEAFAALVTLVGYDWGGVIALALARPEVRGVVVMETHLPPIGKFFGELVLEEEFLA

RSLNGLTDDRDAYYPYRRPMLQWDVVDNAVPVLLVTGGDGVVWARDHPALEVRLAGHFV
PEDAPEEIAAALLDLLGGPD

>AncDhaA7

MHVGLFLHYAEAGTSSPVVFLHGFPESWYVWRHVMTAVAYRCLAPDLRGFGSSDKPAYRL
EDHLEDLHALDAVLVVLVAYDVGGVVALHQARPDVEGVVTLETPLPNKKHMAELFMREKF
VARSFIGLTDDLDVYYPYRAALQLVRAKPALLVTGGDTGVVSAREFPALEEYLAGHFVQEE
APEQITAALLDLLGGHA

>AncDhaA8

MDKHVRGLKLHVAEIGTSVVVFLHGFPEIWYSWRHQMIAVAFRAIAPDYRGYGLSDPPAEK
ARFRDLVSDLVAIDALIVFLVAKDFGARPAYLFALPEVSGVVTLGVPFLPGPEFYIAEDFGRFK
TVVRNVILPWFTEDLDVYGLYFRTALQVPLVPALLIMGEKDYVISVKEFPDLIYLGSHFVQEQ
FPEQVNELILNFLKKHS

>AncDhaA9

LHGVRLHVVQAGPPLVVLLHGFPEFWYGWRQQIPPLAYRVWAPDQRGYNLSDKPRRAYAI
DELVADVIGLAAFVVGHDWGAAVAWHLAHPRLHKLAILNVPHRWYILFQPELFRWWAGRQ
APTFSDADLAQYRVALRSMWIVPTHIIWGVRDAFLAQLCTDLTYLEATHWVQHEEPEEVNE
LLLEFFES

>AncDhaA10

MSEIGTGFPFDPHYVEVLGERMHYVDVGPRDGTPVLFLHGNPTSSYLWRNIIPHVAPSHRCIA
PDLIGMGKSDKPDLAYFFDDHVRYLDAFIEALGLEEVVLVIHDWGSALGFHWAKRHPERVK
GIVCMEFIRPIPTWDEWPEFARETFQAFRTADVGRELVIDQNAFIEGVLPKCVVRPLSEVEMD
HYRAPFLKPVDREPLWRFPNEIPIAGEPANIVALVEAYMNWLHQSPVPKLLFWGTPGVLIPPA
EAARYAESLPNCKTVDIGPGLHYLQEDNPDLIGSEIARWLPGLA

>AncDhaA11

MSEIGTGCPFDPHYVEVLGERMHYVDVGPREGTPVLFLHGNPTSSYLWRNIIPHVAPSHRCIA
PDLIGMGKSDKPDLAYFFDDHVRYLDAFIEALGLEEVVLVIHDWGSALGFHWAKHHPERVK
GIVCMEFIRPFPTWDDWPEFARETFQAFRTADVGRELVLDQNAFIEGVLPKCVVRPLSEVEM
DHYRAPFLKPVSREPLWRFPNEIPIAGEPANITALVEAYMDWLTQSPVPKLLFWGHPGVLIPP
AEAARYAESLPNCKTVDIGPGLHYLQEDNPDLIGSEIARWLPGLA

>AncDhaA12

MSSIGAGCPFDPHYVEVLGSRMHYVDVGPREGTPVLFLHGNPTSSYLWRNIIPHVAPLHRCIA
PDLIGMGKSDKPDLAYRFDDHVRYLDAFIEALGLERVVLVIHDWGSALGFHWAHHHPERVK
GIVFMEFIRPFPTWDDWPEFARETFQAFRTPGVGRELVLDQNAFIEGVLPKCVVRPLSEVEMD
HYRAPFRKPVSRKPLLRFPNEIPIAGEPANITALVEGYLDWLTQSPVPKLLFWGHPGVLIPPAE
AARYAESLPNCKTVDIGPGLHYLQEDNPDLIGREIARWLPGLA

>**Template2** (P0A3G3, 293 positions)

MSEIGTGFPFDPHYVEVLGERMHYVDVGPRDGTPVLFLHGNPTSSYLWRNIIPHVAPSHRCIA
PDLIGMGKSDKPDLDYFFDDHVRYLDAFIEALGLEEVVLVIHDWGSALGFHWAKRNPERVK
GIACMEFIRPIPTWDEWPEFARETFQAFRTADVGRELIIDQNAFIEGALPKCVVRPLTEVEMDH
YREPFLKPVDREPLWRFPNELPIAGEPANIVALVEAYMNWLHQSPVPKLLFWGTPGVLIPPAE
AARLAESLPNCKTVDIGPGLHYLQEDNPDLIGSEIARWLPAL

>AncDhaA13

MSEIGTGFPFDPHYVEVLGERMHYVDVGPRDGTVVLFLHGNPTSSYLWRNIIPHVAPSHRCIA
PDLIGFGKSDKPDLDYFFDDHVRYLDAFIEALGLEEVVLVIHDWGSALGFHWAKRNPERVKG
IACMEFIRPIPTWDEFPEFARETFQKFRTADVGRELIIDQNAFIEGVLPKCVVRPLTEVEMDHY
REPFLKPVDREPLWRFPNELPIAGEPANIVALVEAYMNWLHQSPVPKLFFWGTPGVLIPPAEA
ARLAESLPNCKTVDIGPGLHYLQEDNPDLIGSEIARFLPEL

>AncDhaA14

MSEIGTGFPFDPHYVEVLGERMHYVDVGPRDGTPVLFLHGNPTSSYLWRNIIPHVAPSHRCIA
PDLIGMGKSDKPDLDYFFDDHVRYLDAFIEALGLEEVVLVIHDWGSALGFHWAKRNPERVK
GIACMEFIRPIPTWDEWPEFARETFQKFRTADVGRELIIDQNAFIEGVLPKCVVRPLTEVEMDH
YREPFLKPVDREPLWRFPNELPIAGEPANIVALVEAYMNWLHQSPVPKLLFWGTPGVLIPPAE
AARLAESLPNCKTVDIGPGLHYLQEDNPDLIGSEIARFLPEL

>AncDhaA15

MSEIGTGFPFDPKYVEVLGERMHYVDVGPRDGTVVLFLHGNPTSSYLWRNIIPHVAPSHRCIA
PDLIGMGKSDKPPDLDYFFDDHVRYLDAFIEALGLEEVVLVIHDWGSALGFDWAKRNPERV
AGIACMEFIRPIPTWDEWVEFARETFQAFRDADVGRELIIDQNAFIEGVLPKCVVRPLTEVEM
DHYREPFLKPVDREPLWRFPNELPIAGEPANIVELVEAYMNWLHQSPVPKLFFWGTPGVLIPP
AEAARLAEWLPNCKTVDIGPGLHYLQEDNPHLIGSEIARWLPEL

>AncDhaA16

MSEIGTGFPFDPKYVEVLGERMHYVDVGPRDGTPVLFLHGNPTSSYLWRNIIPHVAPSHRCIA
PDLIGMGKSDKPDLDYFFDDHVRYLDAFIEALGLEEVVLVIHDWGSALGFDWAKRNPERVA
GIACMEFIRPIPTWDEWVEFARETFQAFRDADVGRELIIDQNAFIEGVLPKCVVRPLTEVEMD
HYREPFLKPVDREPLWRFPNELPIAGEPANIVELVEAYMNWLHQSPVPKLLFWGTPGVLIPPA
EAARLAESLPNCKTVDIGPGLHYLQEDNPHLIGSEIARWLPEL

>AncDhaA17

MSEIGTDFPFDPKYVEVLGERMHYVDVGPPDGTPVLFLHGNPTSSYLWRNIIPHVAPSHRCIA
PDLIGMGKSDKMSLDYFFDDHVRYLDAFIEALVLEEVVLVIHDWGSALGLDWAKRNEERVA
GIACMEFIHPIPTWDEWVEFWRENFQAFRDPDVGRELIIDQNLFIEVVLPKYVVRPLTEVEMD
HYRRPFLKPVDREPLWRFPNELPIEGRPANIVELVEKYWNWLHQSPVPKLLFWGTPGVLIPPA
EAARLAEWLPNPKTVDIGPGLHFLQEDNPHLIGSETARWLPEL

>AncDhaA18

MSEIGTDFDFDLHHVEVLGSRMAYVDTGPRDGTVVLFLHGNPTSSYLWRNIIPHVSPSHRCIA
PDLIGFGKSGKPDLDYRFDDHVRYLDAFIEALGLEEVVLVIHDWGSALGFHWAKRNPDRVK
GLAFMEFIRPIPTWDEWPPFARETFRAFRDTPVGRELIIDQNAFIEGALPKCVVRPLSEVEMDH
YRAPFLKPVSREPLWRFPNELPIAGEPANVVALVEKYMNWLHQSPVPKLLFWGTPGVLIPPA
EAARYAETLPNCKTVDLGPGLHYLQEDHPDLIGSEIADWLPEI

>AncDhaA19

MSEISSTFPFEKHYVEVLGSRMTYVDTGPEDTVVLFLHGNPTSSYLWRNIIPHVSPSHRCIAPD
LIGMGDSGKPDLDYFFKDHYRYLDAFIEALGLEEVVLVIHDWGSALGFHWAKRNPDRVVGL
AFMEFIRPPPSWEEWPEFVREMFRAFRDDPLGRELIIDQNFFIEGALPKSVVRPLTEVAMDHY
RAPFLKPVDREPLWRFPNELPIAGEPANVVELVEAYMRFMHETPMPKLLFWGTPGVLIPPAE
AARYAENIPNLKTVYVGPGLHYLQEDNPDLIGREIADWLPRL

>AncDhaA20

MSKPAEIEIRRASVLGSTMAYRETPDAPVALFLHGNPTSSHIWRNILPLVAPVAHCIAPDLIGF
GQSGKPDIAYRFFDHVRYLDAFIEQLGISSAYLVAQDWGTALAFHLAARRPDFVRGLAFMEF
IRPMPTWQDFHQFARETFRKFRTPDVGEAMILEANAFVERVLPGGIVRKLSEEEMAPYRAPFP
TPESRRPVLAFPRELPIAGEPADVYEALQSAHAALAASSYPKLLFTGEPGALVSPEFAERFAAS
LKHCALVRLGAGLHYLQEDHPEAIGRSVAGWIAGIE

9. References

[1] Hon J, Borko S, Stourac J, Prokop Z, Zendulka J, Bednar D, et al. EnzymeMiner: automated mining of soluble enzymes with diverse structures, catalytic properties and stabilities. Nucleic Acids Res 2020;48:W104–9. https://doi.org/10.1093/nar/gkaa372.

[2] Ding X, Zou Z, Brooks III CL. Deciphering protein evolution and fitness landscapes with latent space models. Nat Commun 2019;10:5644. https://doi.org/10.1038/s41467-019-13633-0.

[3] McGee F, Hauri S, Novinger Q, Vucetic S, Levy RM, Carnevale V, et al. The generative capacity of probabilistic protein sequence models. Nat Commun 2021;12:6302. https://doi.org/10.1038/s41467-021-26529-9.

[4] Musil M, Khan RT, Beier A, Stourac J, Konegger H, Damborsky J, et al. FireProtASR: A Web Server for Fully Automated Ancestral Sequence Reconstruction. Brief Bioinform 2021;22:bbaa337. https://doi.org/10.1093/bib/bbaa337.

[5] Lucas JR, Tucker G, Grosse RB, Norouzi M. Don't Blame the ELBO! A Linear VAE Perspective on Posterior Collapse 2019.

[6] Vasina M, Vanacek P, Hon J, Kovar D, Faldynova H, Kunka A, et al. Advanced database mining of efficient haloalkane dehalogenases by sequence and structure bioinformatics and microfluidics. Chem Catal 2022;2:2704–25. https://doi.org/10.1016/j.checat.2022.09.011.

[7] Hon J, Marusiak M, Martinek T, Kunka A, Zendulka J, Bednar D, et al. SoluProt: prediction of soluble protein expression in Escherichia coli. Bioinformatics 2021;37:23–8. https://doi.org/10.1093/bioinformatics/btaa1102.

[8] Mirdita M, Schütze K, Moriwaki Y, Heo L, Ovchinnikov S, Steinegger M. ColabFold: making protein folding accessible to all. Nat Methods 2022;19:679–82. https://doi.org/10.1038/s41592-022-01488-1.

[9] Shroff R, Cole AW, Diaz DJ, Morrow BR, Donnell I, Annapareddy A, et al. Discovery of Novel Gain-of-Function Mutations Guided by Structure-Based Deep Learning. ACS Synth Biol 2020;9:2927–35. https://doi.org/10.1021/acssynbio.0c00345.

[10] Price MN, Dehal PS, Arkin AP. FastTree 2 – Approximately Maximum-Likelihood Trees for Large Alignments. PLOS ONE 2010;5:e9490. https://doi.org/10.1371/journal.pone.0009490.

[11] Sohn K, Lee H, Yan X. Learning Structured Output Representation using Deep Conditional Generative Models. Adv. Neural Inf. Process. Syst., vol. 28, Curran Associates, Inc.; 2015.

[12] Yao Y, Wang X, Ma Y, Fang H, Wei J, Chen L, et al. Conditional Variational Autoencoder with Balanced Pre-training for Generative Adversarial Networks. 2022 IEEE 9th Int. Conf. Data Sci. Adv. Anal. DSAA, 2022, p. 1–10. https://doi.org/10.1109/DSAA54385.2022.10032367.

[13] Ziegler C, Martin J, Sinner C, Morcos F. Latent generative landscapes as maps of functional diversity in protein sequence space. Nat Commun 2023;14:2222. https://doi.org/10.1038/s41467-023-37958-z.

[14] Aslan-Üzel AS, Beier A, Kovář D, Cziegler C, Padhi SK, Schuiten ED, et al. An Ultrasensitive Fluorescence Assay for the Detection of Halides and Enzymatic Dehalogenation. ChemCatChem 2020;12:2032–9. https://doi.org/10.1002/cctc.201901891.

[15] Repecka D, Jauniskis V, Karpus L, Rembeza E, Rokaitis I, Zrimec J, et al. Expanding functional protein sequence spaces using generative adversarial networks. Nat Mach Intell 2021;3:324–33. https://doi.org/10.1038/s42256-021-00310-5.

[16] Anishchenko I, Pellock SJ, Chidyausiku TM, Ramelot TA, Ovchinnikov S, Hao J, et al. De novo protein design by deep network hallucination. Nature 2021;600:547–52. https://doi.org/10.1038/s41586-021-04184-w.

[17] Schenkmayerova A, Pinto GP, Toul M, Marek M, Hernychova L, Planas-Iglesias J, et al. Engineering the protein dynamics of an ancestral luciferase. Nat Commun 2021;12:3616. https://doi.org/10.1038/s41467-021-23450-z.

[18] Buryska T, Vasina M, Gielen F, Vanacek P, van Vliet L, Jezek J, et al. Controlled Oil/Water Partitioning of Hydrophobic Substrates Extending the Bioanalytical Applications of Droplet-Based Microfluidics. Anal Chem 2019;91:10008–15. https://doi.org/10.1021/acs.analchem.9b01839.

[19] Micsonai A, Moussong É, Wien F, Boros E, Vadászi H, Murvai N, et al. BeStSel: webserver for secondary structure and fold prediction for protein CD spectroscopy. Nucleic Acids Res 2022;50:W90–8. https://doi.org/10.1093/nar/gkac345.

[20] Drew ED, Janes RW. PDBMD2CD: providing predicted protein circular dichroism spectra from multiple molecular dynamics-generated protein structures. Nucleic Acids Res 2020;48:W17–24. https://doi.org/10.1093/nar/gkaa296.
[21] Koudelakova T, Chovancova E, Brezovsky J, Monincova M, Fortova A, Jarkovsky J, et al. Substrate specificity of haloalkane dehalogenases. Biochem J 2011;435:345 LP – 354. https://doi.org/10.1042/BJ20101405.
[22] Crooks GE, Hon G, Chandonia J-M, Brenner SE. WebLogo: A Sequence Logo Generator. Genome Res 2004;14:1188–90. https://doi.org/10.1101/gr.849004.
